# Single-cell yolk-shell nanoencapsulation for long-term viability with size-dependent permeability and molecular recognition

**DOI:** 10.1093/nsr/nwaa097

**Published:** 2020-05-09

**Authors:** Li Wang, Yu Li, Xiao-Yu Yang, Bo-Bo Zhang, Nöelle Ninane, Henk J Busscher, Zhi-Yi Hu, Cyrille Delneuville, Nan Jiang, Hao Xie, Gustaaf Van Tendeloo, Tawfique Hasan, Bao-Lian Su

**Affiliations:** State Key Laboratory of Advanced Technology for Materials Synthesis and Processing, Wuhan University of Technology, Wuhan 430070, China; Laboratory of Inorganic Materials Chemistry (CMI), University of Namur, Namur B-5000, Belgium; State Key Laboratory of Advanced Technology for Materials Synthesis and Processing, Wuhan University of Technology, Wuhan 430070, China; State Key Laboratory of Advanced Technology for Materials Synthesis and Processing, Wuhan University of Technology, Wuhan 430070, China; Laboratory of Inorganic Materials Chemistry (CMI), University of Namur, Namur B-5000, Belgium; Namur Research Institute for Life Sciences (Narilis), University of Namur, Namur B-5000, Belgium; Department of Biomedical Engineering, University of Groningen and University Medical Centre Groningen, Groningen 9713 AV, The Netherlands; State Key Laboratory of Advanced Technology for Materials Synthesis and Processing, Wuhan University of Technology, Wuhan 430070, China; Nanostructure Research Centre (NRC), Wuhan University of Technology, Wuhan 430070, China; Laboratory of Inorganic Materials Chemistry (CMI), University of Namur, Namur B-5000, Belgium; State Key Laboratory of Advanced Technology for Materials Synthesis and Processing, Wuhan University of Technology, Wuhan 430070, China; School of Engineering and Applied Sciences, Harvard University, Cambridge, MA 02138, USA; State Key Laboratory of Advanced Technology for Materials Synthesis and Processing, Wuhan University of Technology, Wuhan 430070, China; Nanostructure Research Centre (NRC), Wuhan University of Technology, Wuhan 430070, China; Electron Microscopy for Materials Science (EMAT), University of Antwerp, Antwerp B-2020, Belgium; Cambridge Graphene Centre, University of Cambridge, Cambridge CB3 0FA, UK; State Key Laboratory of Advanced Technology for Materials Synthesis and Processing, Wuhan University of Technology, Wuhan 430070, China; Laboratory of Inorganic Materials Chemistry (CMI), University of Namur, Namur B-5000, Belgium

**Keywords:** cell surface engineering, protein internalization, ordered colloidal packing, high photosynthetic ability, harsh condition resistance, thiol-functionalization

## Abstract

Like nanomaterials, bacteria have been unknowingly used for centuries. They hold significant economic potential for fuel and medicinal compound production. Their full exploitation, however, is impeded by low biological activity and stability in industrial reactors. Though cellular encapsulation addresses these limitations, cell survival is usually compromised due to shell-to-cell contacts and low permeability. Here, we report ordered packing of silica nanocolloids with organized, uniform and tunable nanoporosities for single cyanobacterium nanoencapsulation using protamine as an electrostatic template. A space between the capsule shell and the cell is created by controlled internalization of protamine, resulting in a highly ordered porous shell-void-cell structure formation. These unique yolk-shell nanostructures provide long-term cell viability with superior photosynthetic activities and resistance in harsh environments. In addition, engineering the colloidal packing allows tunable shell-pore diameter for size-dependent permeability and introduction of new functionalities for specific molecular recognition. Our strategy could significantly enhance the activity and stability of cyanobacteria for various nanobiotechnological applications.

## Teaser

Single-cell yolk-shell nanoencapsulation endows living cells with a hierarchical ordered porous structure, significantly enhancing biological activity, stability, molecular recognition and resistance to harsh environment for various nanobiotechnological functionalisation and applications.

## INTRODUCTION

Single-cell nanoencapsulation is an emerging, non-genetic technique to create extended cell surface functionalities, provide external stimuli to enhance cell stability and activity, and incorporate new properties usually available only through genetic modification [1–4]. Compared to multi-cell encapsulation [5], single-cell nanoencapsulation allows cell density control, cell behaviour monitoring, cell characterization on single-cell level and the control of cell localization. Such encapsulation method facilitates the mass and the light penetration from the exterior environment to encapsulated cells, avoids cell aggregation and decreases the amount of encapsulating materials. Last but not least, it also allows the functionalization of external surface to confer new functionalities to the system, thus making it a promising tool for fundamental investigation on single-cell level and contributing to higher efficiency and cost-saving in applications. Furthermore, single-cell capsules offer a new model for the design of engineered living materials which are constructed from living systems [6–8] and show a large range of applications, including adhesion glues [9], biocatalysis [10] and biodegradation [11]. The most commonly employed encapsulants include silica [12–14], metal-organic frameworks [15], calcium phosphates [16] and gold [17,18], as well as materials with specific functionalities, such as stimulating cadmium sulphide [19,20], thermally stable metal oxides [21], light-harvesting indium phosphide [22] and conductive carbon [23]. Colloidal packing is a common strategy for single-cell encapsulation through an adsorption–assembly–encapsulation sequence [24]. However, the complexity and fluidity of the surface of the cells [25] make it challenging to accurately control the encapsulation structure and shell permeability around individual cells. The disordered structure of current generation colloidal packings does not allow well-controlled exchange between a cell and its environment, affecting nutrient, waste and metabolite diffusion and therefore cell activity and stability. Moreover, such colloidal packing results in direct contact between the shell and the cell surface [26–29]. This is incompatible with cells and negatively affects their activity and stability even further.

The global market for microbes and microbial products was valued at US$170.5 billion in 2017 and is projected to approach nearly US$302.7 billion in 2023 [30]. Currently, many applications of bacteria in advanced biotechnology and bio-energetics are impeded by low biological activity and stability in industrial reactors. Therefore, enhancing bacteria activity and stability not only presents a significant scientific and technological breakthrough but also has a strong potential to cater for market requirements.

Human progress has long benefited from natural as well as synthetic materials inspired by nature. For example, in an attempt to protect and preserve structures of nanomaterials, yolk-shell configuration, with a void between a well-controlled porous shell and fragile core, was developed by mimicking the structures of eggs [31–34]. The yolk-shell structure of eggs is the consequence of evolution by natural selection and offers a biocompatible, physiological environment to the cell. Such a natural interfacing scheme could be used as a model to develop novel cell encapsulation technologies. The detrimental, direct contact between the cell surface and the shell in the conventional cell encapsulation strategy can be avoided by using a buffer to create a biocompatible environment in the interstitial voids to cells. Consequently, single cells encapsulated in yolk-shell configuration with structural superiority could successfully retain their biological activity, possess highly enhanced stability and photosynthetic activity and significantly extended functionalities as required in advanced biotechnological applications such as photobioreactors, bioelectronics, biocatalysis, biosensors and biofuel reactors [35–37].

It is well known that the ordering at microscopic and nanoscopic scale provides living organisms with greater environmental adaption and communication abilities, which has resulted in the evolution of unicellular organisms into multicellular organisms [38–40]. Therefore, the degree of structural ordering of cell surfaces serves as an evolutionary marker. For example, ordered structures at the microscopic scale on the surface of archaea cells aid their stability in hostile environments [41], which is believed to be the reason why this organism appears on the evolutionary timeline of eukaryotes just after the emergence of bacteria [42]. Surface porosity is one of the most important microscopic and nanoscopic scale features of advanced cellular structure [43], such as the uniform inorganic pores of Foraminifera [44] or the tunable hybrid pores of embryophytes for mass-energy-information exchange and storage [45], offering not only excellent control for nutrient uptake and waste excretion and protection in harsh conditions, but also size selectivity to different species. Some recent and very successful attempts have been made toward the construction of ordered mesoporous structures around living cells through multi-cell encapsulation by a 3D lipid-silica matrix or by glycerol-doped silica gel monoliths [46–48]. The ordered mesoporosity of the 3D matrix and the presence of the interstitial lipid layer leads to enhanced viability of the entrapped multi-cells [46], which could enable long-term preservation of living cells. This successful 3D, conformal encapsulation technology within ordered lipid-silica mesophases or glycerol-doped silica gel monoliths can, however, only be used for multi-cell encapsulation and is at present challenging to apply to single cells.

Here we report highly stable and long-lived single cyanobacterium capsules with an ordered yolk-shell structure of uniformly organized and tunable nanoporosity shaped by protein-assisted, hydrophilic colloidal silica packing. The void between the ordered nanoporous shell and cell is created by a precisely- and time-controlled internalization of protamine, which could subsequently be filled by nutrients. This is the first experimental evidence of internalization of protamine into the cell cytoplasm and is of great scientific importance for the development of future cell-encapsulation strategies. Shells thus constructed are not only biocompatible but also endow introduction of new and unprecedented cell surface functionalities, such as specific size-dependent permeability and defined molecular recognition abilities. Owing to the presence of the buffering interstitial hollow space filled by nutrient between the ordered nanoporous shell and the cell surface, cyanobacterial activity and stability evolving from this yolk-shell encapsulation technology are highly enhanced. The photosynthetic activity under a large range of photon flux density (0–500 μE m^−2^ s^−1^) is 3 to 4-fold higher than can be obtained by state-of-the-art results where the shells directly contact the encapsulated cyanobacteria. Because of its specific size-dependent permeability stemming from uniformly organized nanoporosity, the survival ability of yolk-shell encapsulated cyanobacteria against toxic chemical environments is significantly strengthened: 5.9, 2.3 and 10-fold higher for 10 nm silver nanoparticles (a potent antimicrobial agent), 6, 1.4 and 70 to 80-fold higher for polydiallyldimethylammonium chloride (PDADMAC, an antimicrobial molecule), and 2, 40 and 7-fold higher for octane than cells encapsulated in disordered shells, cells directly contacting the encapsulating shells and native cyanobacteria, respectively. In addition, the ordered yolk-shell structure can also provide encapsulated cyanobacteria with superior protection against some harsh biological or physical conditions, such as the resistance against lysozyme or thermoprotection.

Protamine is an arginine-rich protein in which each arginine side chain (NHC(NH_2_) = NH_2_^+^) possesses a positive charge at its centre [49]. This feature enables the self-assembly of protamine on the cell surface through electrostatic binding with amply available, negatively charged cell surface components [50]. Another attractive property of protamine is that it may be internalized into the cytoplasm of cells although no direct experimental evidence of this internalization exists [51–53]. Protamine is thus chosen to assist well-ordered silica colloidal packing through its self-assembly on the cell surface and importantly, to investigate the possibility of creating a void between the cell and the shell upon the possible internalization of protamine into the cells. Different reports have implicated the importance of the interface chemistry between the cell surface and the encapsulating host matrix [5,14,16,46–48,54,55]. The regular backbone structure of protamine could act as a structure-directing agent to generate a well-organized silica colloidal packing [56] and facilitate the control of the internalization of protamine in the cell [51–53]. Through experimental demonstration of precisely- and time-controlled kinetics of protamine internalization, we successfully create the voids between the cell surface and the ordered colloidal packing. The space created and subsequently filled by nutrients offers a high buffering capability to cells and a much more favourable biocompatible environment for their enhanced stability and activity. As a comparison, polyethylenimine (PEI) has also been investigated, indicating that it can only be adsorbed on the cell surface of cyanobacteria without any internalization happening. The single-cell encapsulation technology developed in this work, allowing the construction of ordered and buffered shells with well-controlled and uniformly organized nanoporosity can offer structural superiority, performance enhancement and potential extension to a range of applications.

## RESULTS

### Single-cell yolk-shell encapsulation

Cyanobacteria are of great importance in bio-nanotechnology [5,35,36] and bio-energetics [57] because of their simple cellular structure and high photosynthesis conversion efficiency. However, they often suffer from reduced viability in hostile environments. Therefore, cyanobacterium *Synechocystis sp. PCC 7002* (Supplementary Figs 1a–c and 2) was chosen as the model cell for our study.

**Figure 1.**
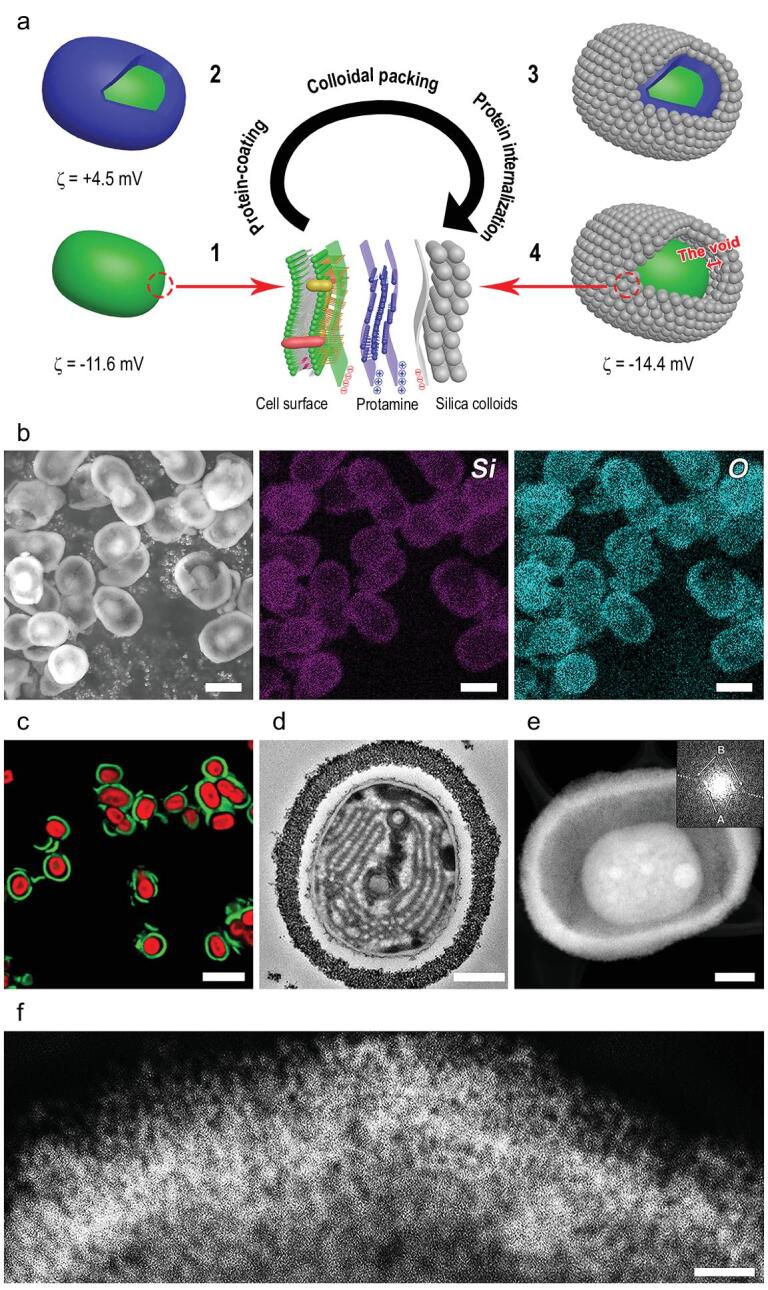
The formation of yolk-shell single-cell capsules. (a) Schematic illustration of sequential steps in the protamine-assisted colloidal packing for encapsulation of cyanobacterium *Synechocystis sp. PCC 7002*, including the zeta potentials (ζ) of the encapsulated bacteria suspended in 10 mM PBS (pH 7.4) after each sequential step: (i) a native cyanobacterium, (ii) a protamine-coated cyanobacterium, (iii) the colloidal packing onto adsorbed protamine layers on a cyanobacterium, (iv) the internalization of protamine to create a void between the colloidal packing layer and the cell surface. All figures are made with a single-cell capsule synthesized with colloidal nanoparticles of 14.9 nm in diameter. (b) SEM micrograph of the cyanobacteria encapsulated in a protamine-assisted, ordered silica shell. EDX mapping for elemental Si and O are also shown, confirming the presence of the silica shell. Scale bars: 2 μm. (c) Merged CLSM micrograph of encapsulated cyanobacteria, showing individually encapsulated, red fluorescent cyanobacteria and green fluorescent silica shells with interstitial voids between the cyanobacteria and the shells. Scale bar: 5 μm. (d) TEM micrograph of an encapsulated cyanobacterium, showing intracellular structures and the colloidal packing layer. Scale bar: 500 nm. (e) HAADF-STEM micrograph and FFT (inset) image of an encapsulated cyanobacterium. The FFT image indicates order of the colloidal packing. Scale bar: 500 nm. (f) HAADF-STEM micrograph of the colloidal packing layer. Scale bar: 100 nm.

Our synthesis method for single-cell nanoencapsulation with an ordered yolk-shell structure with uniformly organized nanoporosity consists of three distinct steps as schematically illustrated in Fig. 1a. Native cyanobacterium *Synechocystis sp. PCC 7002* typically possesses a negative charge, with a zeta potential of −11.6 mV. After self-assembly of positively charged protamine on the cyanobacterial cell surface, the zeta potential increases to +4.5 mV. It is found that the zeta potential of the protamine-coated cyanobacteria returns to −11 mV within 1 h, close to the zeta potential of the native cyanobacteria. This change in the zeta potential from the initial negative value of −11.6 mV to the final negative value of −11 mV, through an intermediate positive value of +4.5 mV strongly indicates the internalization of protamine into the cytoplasm of cyanobacteria immediately after its self-assembly at the surface of cyanobacteria. Thus, to ensure the interaction of the positively charged guanido-terminated residual arginine and amino-terminated residual lysine of the adsorbed protamine with the negatively charged silica nanoparticles to direct their packing, the silica nanoparticles should be added immediately following protamine self-assembly. This allows the formation of an ordered silica nanoparticle packing structure with a uniformly organized nanoporous shell around the protamine-coated cells (Fig. 1a). At the same time, the internalization of protamine into the cytoplasm of cyanobacteria can start, eventually leading to the formation of the well-ordered shell-void-cell structure, with a zeta potential of −14.4 mV. The successful encapsulation in this process is dependent on a precisely followed protocol, matching the kinetics of the internalization of protamine.

Scanning electron micrographs (SEM) and energy-dispersive X-ray (EDX) analysis show the bare surface of the native cyanobacteria (Supplementary Fig. 1a–c) and the presence of silica shells on the single-cell yolk-shell encapsulated cyanobacteria (Fig. 1b and Supplementary Fig. 1d–f). Confocal laser scanning microscopy (CLSM) confirms the encapsulation of single cyanobacteria and demonstrates the presence of interstitial voids, characteristic of a yolk-shell structure (Fig. 1c). The difference in surface structure between the native and encapsulated cyanobacterium observed by transmission electron microscopy (TEM, Fig. 1d and Supplementary Fig. 2) confirms the presence of interstitial voids in the encapsulated cyanobacterium and suggests that the internalization of protamine into the cyanobacterium occurs to create the voids. High-angle annular dark-field scanning transmission electron micrograph (HAADF-STEM, Fig. 1e) gives another clear illustration of the yolk-shell encapsulated cyanobacterium. The thickness of the silica colloidal packing layer and the width of the interstitial voids is estimated from the TEM micrographs (Fig. 1d) to be 200 and 100 nm, respectively. We note that the difference in the width of the interstitial voids observed by TEM and HAADF-STEM techniques results from the difference in sample preparation (see Methods). Very importantly, the corresponding Fast Fourier Transform (FFT) (inset to Fig. 1e) shows a long-range periodicity of the order of the silica nanoparticles in the shell. High magnification imaging (Fig. 1f) again shows the radially outward ordered structures. In the innermost part of the shells, nanoparticles are densely packed (Fig. 1f and Supplementary Fig. 3), but disordered due to the fluidity [25] and curvature [58] of the cell surface and localized effects of the high protein charges [59]. The transition from a disordered packing in the inner shell to the ordered packing in the outer shell is due to the colloidal freezing transition from densely packed aggregation to spontaneous crystallization in which colloidal nanoparticles have the freedom to arrange themselves into an ordered structure [60]. Therefore, the packing pattern of nanoparticles is a long-range periodicity of the order. The formation of such ordered silica packing with a long range periodicity and uniformly organized nanoporosity at the outer layers of the shell is evidenced by the FFT of the STEM image. This is of critical importance to tailor the porosity of the shell which permits precise control of the penetration or diffusion of nutrients, oxygen, wastes and metabolites, while offering a protective barrier and specific size selectivity against hostile environments.

To confirm the biocompatibility of our encapsulation protocol, the cytotoxicity of protamine, as well as another intermediate layer-constructing candidate PEI, on cyanobacteria was evaluated. We found that at low concentration (0.1 mg mL^−1^, the concentration used for cell encapsulation), protamine and PEI did not negatively affect the viability of cyanobacteria (∼85% in the protamine treatment (Supplementary Fig. 4a), ∼80% in the PEI treatment (Supplementary Fig. 4e)). However, for increased concentration (0.2, 0.5 and 1.0 mg mL^−1^), the cell viability gradually decreased (Supplementary Fig. 4b–d and 4f–h), underscoring the negative effect of protamine and PEI at high concentrations. Please note that the encapsulating shells formed using protamine with the concentrations lower than 0.1 mg mL^−1^ could not be uniform, intact and ordered around the cyanobacteria (Supplementary Fig. 5). When protamine is not enough to coat the whole cyanobacterial cell surface, silica nanoparticles can only deposit on the cell surface covered by protamine and cannot deposit on the rest of the bare cell surface due to the repulsion generated by the negative charge on silica nanoparticles and cell surface. In addition, the protamine with a low concentration cannot show enough power to drive the assembly of silica nanoparticles into an ordered structure.

### Internalization behaviour of protamine

For the yolk-shell encapsulation, in addition to priming the cell surface to induce colloidal packing to form a shell, an intermediate layer formed by the self-assembled protamine molecules should also offer the possibility of being internalized by the cells to create a void between the cell surface and the shell. Our results above, showing the formation of the yolk-shell structure with a void between the cell surface and the ordered silica nanoparticles packing shell, are attributed to the possible internalization of protamine molecules into the cells. However, the experimental evidence on the creation of such voids between the cell and shell by internalization of protamine has never been reported. Therefore, the internalization behaviour of two intermediate layer-constructing candidates, protamine and PEI, into the cytoplasm of cyanobacteria was studied.

**Figure 2.**
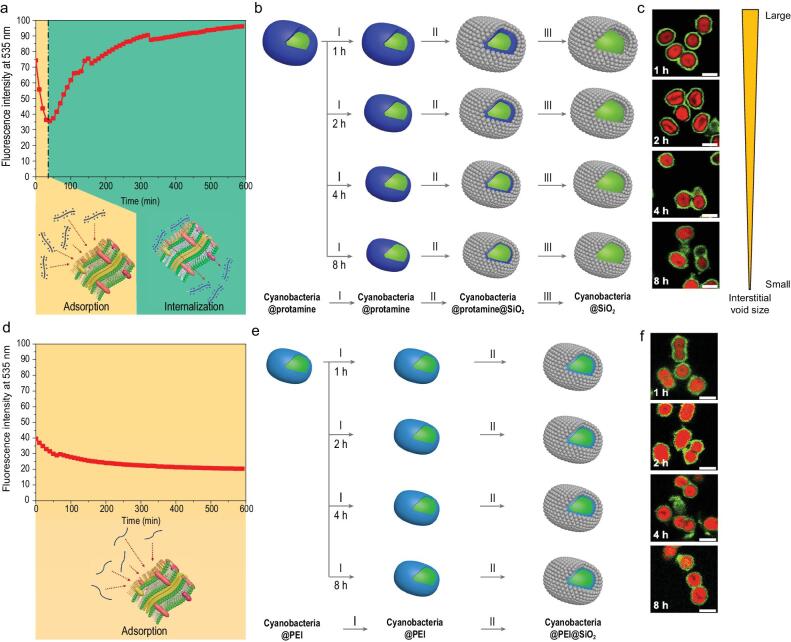
Internalization behaviour of cyanobacteria and the corresponding effects on the yolk-shell encapsulation. (a) Fluorescence intensity at 535 nm of the cyanobacteria-protamine mixture over time obtained by *in situ* fluorometry spectroscopic study. The decrease in fluorescence intensity indicates the adsorption of protamine on the cell surface (yellow colour), whereas the increase indicates the internalization of protamine into the cytoplasm (green colour). (b) Schematic illustration of sequential steps for silica packing around the protamine (blue colour)-coated cyanobacterium which had been incubated in the medium for 1, 2, 4 and 8 h before the addition of silica colloids into suspension. The yolk-shell formation follows three steps: (I) incubation, (II) colloidal packing and (III) protein internalization. The internalization can occur during step (I). (c) Merged confocal laser scanning microscopy (CLSM) micrographs of the encapsulated cyanobacteria whose silica shells are fluorescence-labelled show individually encapsulated cyanobacteria: red fluorescent cyanobacteria and green fluorescent silica shells. Scale bars: 2 μm. The size of the void between the silica shell and the cell surface decreases with the prolonged incubation time of the protamine-coated cyanobacteria as the internalization can occur before the addition of silica colloidal nanoparticles. (d) Fluorescence intensity at 535 nm of the cyanobacteria-PEI mixture with time obtained by an *in situ* fluorometry spectroscopic study. The decrease in fluorescence intensity with incubation time to reach a plateau indicates clearly that only the adsorption of PEI on the cell surface occurs (yellow colour). (e) Schematic illustration of the sequence of steps for silica packing around the PEI (cyan colour)-coated cyanobacterium which had been incubated in the medium for 1, 2, 4 and 8 h. The silica coating follows only two steps: (I) incubation and (II) colloidal packing. (f) Merged CLSM micrographs of the encapsulated cyanobacteria whose silica shells are fluorescence-labelled show individually encapsulated cyanobacteria: red fluorescent cyanobacteria and green fluorescent silica shells. Scale bars: 2 μm. There is no void between the silica shell and the cell surface in the PEI-mediated silica-encapsulating cyanobacteria.

The behaviour of the protamine and the PEI at the surface of cyanobacteria was investigated by tracking the fluorescent intensity (at 535 nm) of the fluorescent dye (pHrodo™ iFL Green STP Ester)-labelled protamine (Fig. 2a) and PEI (Fig. 2d) over time using *insitu* fluorescence spectroscopy. In both cases, the fluorescence is expected to dramatically increase if the internalization occurs as the pH value of the medium changes from basic to acidic due to the acidification of intracellular vesicles. In our experiment, the fluorescence intensity of the cyanobacteria-protamine mixture decreased at the beginning of the incubation (0–40 min). This is attributed to the adsorption of the dye-labelled protamine on the surface of the cyanobacteria. Subsequently (40–600 min), an increase in the fluorescence intensity is observed. This can be attributed to the internalization of the protamine from the cell surface to the cytoplasm (Fig. 2a). On the other hand, the fluorescence intensity of the cyanobacteria-PEI mixture decreased gradually until reaching a plateau, demonstrating that only PEI adsorption occurred during the incubation without any internalization into cyanobacteria (Fig. 2d). It is worth noting that PEI internalization into the cell may take place for other types of cells. Even though PEI cannot be internalized into the cytoplasm of cyanobacteria, it can easily be internalized by microalgae cells if their cell walls are removed. The internalization behaviour of the fluorescence-labelled PEI into the cell wall-removed microalgae cells is evidenced by an increase of fluorescence intensity with incubation time, in particular, at the beginning of contact between cell wall-removed microalgae cells and fluorescence-labelled PEI (Supplementary Fig. 6). Please note that owing to the rapid PEI internalization of the microalgae cells, only the increase of the fluorescence intensity could be observed. This change in fluorescence intensity is opposite to that observed in the fluorescence-labelled PEI in contact with cyanobacteria. This confirms that although PEI molecules could be internalized by the microalgae cells without cell walls, they could only be adsorbed at the surface of cyanobacteria. In addition, the internalization behaviours of cyanobacteria was further confirmed by the observation of fluorescent dye (5(6)-carboxyfluorescein *N*-hydroxysuccinimide ester)-labelled protamine and PEI over time using CLSM. In CLSM micrographs (Supplementary Fig. 7), fluorescent dye-labelled protamine and PEI show green fluorescence, while cyanobacteria show red fluorescence because of the presence of *Chlorophyll a*. After 0.5-h incubation, most protamine were observed around cyanobacteria, indicative of the initial adsorption. With increasing the incubation time, fluorescent dye-labelled protamine can be seen inside cyanobacteria, indicating the occurrence of the internalization of protamine. After 4.5-h incubation, a large part of cyanobacteria presented internalized fluorescent dye-labelled protamine in their cytoplasm as indicated by the arrows in the CLSM micrographs. Whereas, fluorescent dye-labelled PEI molecules were observed around cyanobacteria during the whole incubation time with some formed aggregates outside the cyanobacteria (bright green spots as shown in the CLSM micrographs), evidencing that no internalization of PEI occurred. It is also worth noting that silica nanoparticles cannot be internalized by cyanobacteria which can be proven by the fact that none of the silica nanoparticles are observed in the cytoplasm of the cyanobacterium (Supplementary Fig. 3a). Given the above results obtained using two different labelling green fluorescent dyes, we therefore propose that unlike PEI, protamine molecules can be internalized into the cytoplasm of cyanobacteria, which is essential to the success of our strategy for the formation of the single-cell yolk-shell capsules.

The time-controlled internalization is critical to the success of our single-cell nanoencapsulation strategy. The yolk-shell structure formation kinetics were studied to choose the optimum moment for adding silica nanoparticles and for their assembly to form the highly ordered porous silica shell. Figure 2b and e schematically illustrate the different steps of the internalization of protamine (Fig. 2b) and the adsorption of PEI (Fig. 2e). For this experiment, we prepared protamine-adsorbed cyanobacteria with a range of incubation times (1, 2, 4, 8 h), representing different states of internalization. Subsequently, silica nanoparticles were deposited on the surface of these protamine-coated cyanobacteria for cell encapsulation (Fig. 2b). The silica shells of the encapsulated cyanobacteria were stained with pHrodo^TM^ Green and characterized using CLSM. The CLSM micrographs (Fig. 2c) present a cell-in-shell structure, with interstitial voids that can be clearly seen in the encapsulated cyanobacteria with 1 and 2 h incubation pre-treatment. Smaller interstitial voids can also be seen in the encapsulated cyanobacteria with 4 h incubation pre-treatment. However, the interstitial voids could be hardly observed in the encapsulated cyanobacteria with 8 h incubation pre-treatment. With the prolongation of the incubation time of the protamine-coated cyanobacteria, the formed interstitial voids between the cyanobacteria and the silica shells became gradually smaller (Fig. 2b and c).

According to the internalization kinetics of protamine presented in Fig. 2a, the surface-adsorbed protamine is internalized into the cytoplasm as the incubation time increases, reducing the amount of protamine adsorbed on the cell surface. Based on this observation, we propose that the thickness of the adsorbed protamine during the shell formation defines the size of the interstitial voids created by the internalization of protamine. Therefore, constructing silica shells around the cyanobacteria before the internalization of the adsorbed protamine molecules into the cytoplasm is the key to controlling the cell encapsulation process. The most appropriate moment to add nanoparticles for cell encapsulation is immediately after the self-assembly of protamine. On this basis, the yolk-shell encapsulation using protamine molecules was carried out. Supplementary Fig. 8 shows SEM and large zone low magnification TEM micrographs of yolk-shell single-cell capsules synthesized by adding silica nanoparticles of different diameters. This leads to the formation of silica packing with defined pore size in the ordered shells as soon as the protamine molecules are introduced into the cyanobacteria suspension. These micrographs demonstrate the presence of the void between the cell surface and the silica nanoparticle packed shell for successful yolk-shell formation, and underscore the importance of the study on the internalization kinetics of protamine.

The same study was carried out with the PEI molecules. CLSM micrographs of the control sample with PEI-mediated silica encapsulating cyanobacteria (Fig. 2f and Supplementary Fig. 9b) indicate the absence of such voids regardless of the incubation period. The direct contact between the cell surface and the shell can be evidenced by SEM and TEM (Supplementary Fig. 9a, c and d), regardless of the thickness of shells (Supplementary Fig. 10). This observation is also supported by the unchanged fluorescence intensity after PEI adsorption (Fig. 2c), suggesting that the surface state does not change with incubation time. We therefore conclude that although PEI could be adsorbed on to the cell surface to assist the construction of the silica shell, it could not be internalized into the cytoplasm to induce the formation of voids. The encapsulated cyanobacteria with the PEI-mediated silica shells were termed as encapsulated cyanobacteria with cell-contacting shells and were used as a comparison sample in our study. Another sample concerning cyanobacteria encapsulated in a disordered shell was also prepared (see Methods and Supplementary Fig. 11).

### Tuning the yolk-shell porosity

**Figure 3.**
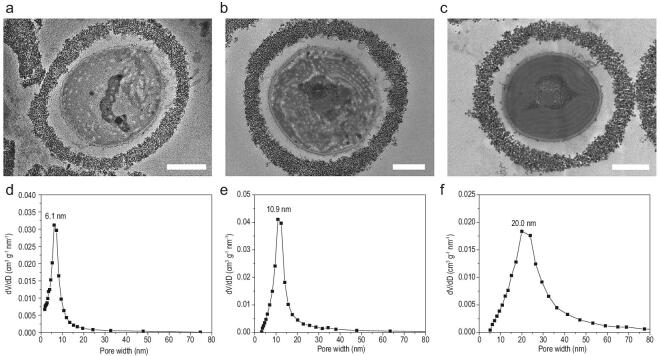
Pore-size tunability. (a–c) TEM micrographs of cyanobacteria encapsulated by protamine-assisted colloidal packing with nanoparticles of different diameters of (a) 9.2, (b) 14.9 and (c) 31.0 nm. Scale bars: 500 nm. (d–f) Pore-size distributions of colloidal packing layers with nanoparticles of different diameters of (d) 9.2, (e) 14.9 and (f) 31.0 nm, determined from analysis of nitrogen adsorption–desorption isotherms, according to Barrett-Joyner-Halenda (BJH) method.

To tune the porosity of the colloidal packing layer, highly monodispersed nanoparticles with different diameters (9.2, 14.9 and 31.0 nm; see Supplementary Fig. 12 and Table 1 for diameter distribution) were used. The colloidal packing layers formed during the encapsulation procedure increase in thickness with increasing nanoparticle diameter, as can be seen in Fig. 3a–c. Single-cell yolk-shell capsules with an ordered colloidal packing and highly uniform nanoporosity can be successfully constructed using silica nanoparticles of different diameter (Supplementary Figs 8 and 12), allowing us to tune the pore sizes (Fig. 3d–f).

The thickness of the shell obtained with silica nanoparticles of 9.2, 14.9 and 31.0 nm (Supplementary Fig. 12) are about 120, 250 and 300 nm, respectively (Supplementary Fig. 8d–f and Table 1). The resultant pore-size distributions of the shell obtained with silica nanoparticles of 9.2, 14.9 and 31.0 nm (Supplementary Fig. 12) are centred at 6.1, 10.9 and 20.0 nm, respectively (Fig. 3d–f). The narrow pore-size distributions in the shells support our conclusion that the nanoparticles in the colloidal packing layer form an ordered structure, with uniform and tunable pore sizes.

### Viability and photosynthetic activity

**Figure 4.**
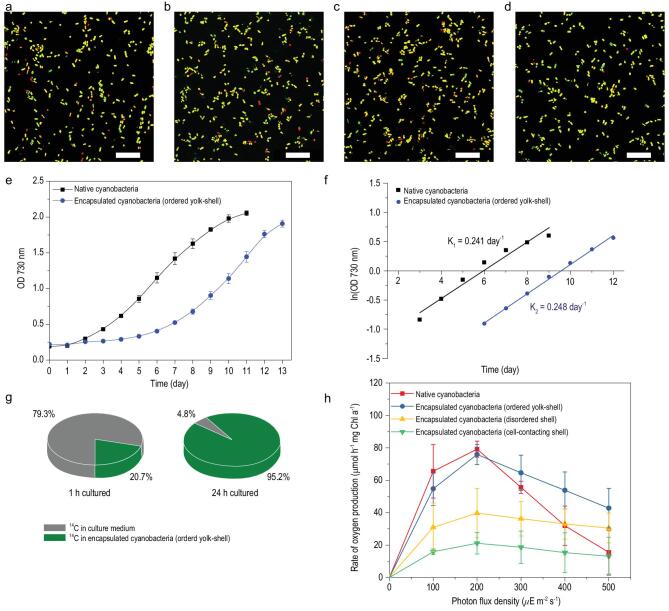
The viability and photosynthetic activity. (a–d) Merged CLSM micrographs of FDA stained non-encapsulated (a), cyanobacteria after encapsulation in ordered yolk-shells with a pore size of 6.1 (b), 10.9 (c) and 20.0 nm (d). Greenish-yellow fluorescent bacteria are alive, while the ones showing red fluorescence are dead. Scale bars: 25 μm. (e, f) Optical densities (OD) at 730 nm of native cyanobacteria and those encapsulated in a hierarchically ordered yolk-shell (pore size of 10.9 nm), in growth medium as a function of time, plotted linearly (e) and logarithmically (f). Error bars indicate standard deviations over three independent cultures. (g, h) Photosynthetic activities of cyanobacteria, measured by carbon fixation (g) and oxygen production (h). Carbon fixation involves uptake of ^14^C from culture medium by cyanobacteria encapsulated in ordered yolk-shell with 10.9 nm pore size after 1 and 24 h of growth. The measured rate of oxygen production of native cyanobacteria, and cyanobacteria encapsulated in ordered yolk-shells (pore size of 10.9 nm), in disordered shells and in cell-contacting shells. The rate of oxygen production is presented as a function of the photon flux density. Error bars indicate standard deviations over three independent measurements with separate cyanobacterial cultures.

CLSM micrographs of fluorescein diacetate (FDA)-stained non-encapsulated cyanobacteria and protamine assisted, encapsulated cyanobacteria show predominantly green fluorescence, indicative of live cyanobacteria (Fig. 4a–d). We find that the percentage of live cyanobacteria is from 92% to 95% for yolk-shell encapsulation induced by protamine, 92% for a disordered shell encapsulation and only 68% for cell contacting shell encapsulation (see Table 1 and Supplementary Fig. 13). Although the shell affects the growth kinetics of the cells by increasing the lag-time (Fig. 4e and Supplementary Fig. 14a), the growth rates of the non-encapsulated and encapsulated cyanobacteria show the same trend (Fig. 4f and Supplementary Fig. 14b). Please note that there is a slight difference in the lag-time of the yolk-shell encapsulated cyanobacteria with different pore size (Supplementary Fig. 14a). The shells turned to be thicker with the increase of the shell pore size (Supplementary Fig. 8 and Table 1), thus the lag-time of the encapsulated cyanobacteria became slightly longer. Besides, very similar visible spectra with fluorescence peaks at 689 nm due to *Chlorophyll a* and at 630 nm due to *phycocyanin* (Supplementary Fig. 15) suggest that cyanobacteria remain unaffected, function normally in our ordered nanoporous yolk-shell capsules, and exhibit the same biological activity and fluorescence as native cyanobacteria.

**Table 1.** Survival ability of cyanobacteria in different toxic chemical environments. Viability of native cyanobacteria and cyanobacteria encapsulated in ordered yolk-shells with different pore size, in disordered shells and in cell-contacting shells after 1 day in the culture medium, with or without supplementation with silver nanoparticles, PDADMAC or octane. Viability was expressed as the percentage of live cyanobacteria, visualized after FDA staining and observed using CLSM. All experiments were carried out with three separate samples with separately cultured cyanobacteria. The data represent percentage averages ± standard deviations obtained after counting a total of 200 cyanobacteria for each encapsulation and medium supplementation.

	Viability (%) at different toxic chemical environments			
Encapsulation	None	Silver nanoparticles	PDADMAC	Octane
Native cyanobacteria	99 ± 1	8 ± 4	1 ± 1	12 ± 8
Yolk-shell, pore size 6.1 nm	95 ± 4	80^a^ ± 4	77^a^ ± 8	81^a^ ± 7
Yolk-shell, pore size 10.9 nm	94 ± 3	83^a^ ± 7	75^a^ ± 2	76^a^ ± 5
Yolk-shell, pore size 20.0 nm	92 ± 4	54^a^ ± 9	74^a^ ± 5	85^a^ ± 5
Disordered shell	92^a^ ± 1	14^b^ ± 5	13^b^ ± 6	39^a,b^ ± 3
Cell-contacting shell	68^a,b^ ± 3	35^a,b^ ± 4	55^a^ ± 8	2^a,b^ ± 2

^a^Significantly different from native cyanobacteria for each medium supplementation at *P* < 0.05 (ANOVA). ^b^Significantly different from cyanobacteria encapsulated in ordered yolk-shell for each medium supplementation at *P* < 0.05 (ANOVA).

Photosynthetic activities of the cyanobacteria after encapsulation were assessed from carbon fixation and oxygen production of the cyanobacteria. Carbon fixation was demonstrated by tracking the transport of ^14^C labelled NaH^14^CO_3_ from a culture medium into the encapsulated cyanobacteria [61]. After 1 h, 20.7% of all ^14^C initially present in the culture medium was found inside the bacteria and after 1 day of culture, 95.2% of all ^14^C was intracellularly found (Fig. 4g). This proves that the ordered yolk-shell structure allows natural diffusion of small inorganic salt molecules through its uniform-sized nanopores, including cyanobacterial carbon sources, maintaining high cyanobacterial viability and activity. A comparison between the rate of oxygen production of native cyanobacteria, and cyanobacteria encapsulated in ordered yolk-shells, in disordered shells, and in cell-contacting shells is then carried out (Fig. 4h). The concentration of *Chlorophyll a* in all experimental cyanobacteria is almost the same (Supplementary Fig. 16). The rate of oxygen production of the ordered yolk-shell encapsulated cyanobacteria was found to be similar to that of the native cyanobacteria at lower photon flux densities and, importantly, highly enhanced (up to 250%) at photon flux densities above 300 μE m^−2^ s^−1^ (significant at *P* < 0.05; ANOVA). The decrease of the oxygen production rate at the high photon flux densities is attributed to the photoinhibition phenomenon. The photoinhibition is light-induced reduction in the photosynthetic capacity of a plant, alga or cyanobacterium. The silica shell could alleviate the photoinhibitory effects under high light stress to maintain the high oxygen production rate of the ordered yolk-shell encapsulated cyanobacteria [62]. The void between cyanobacteria and silica shell can also play a buffer in reducing the light stress. The photosynthetic activity over a large range of photon flux density (0–500 μE m^−2^ s^−1^) (Fig. 4h) is 3 to 4-fold higher than that which can be obtained by state-of-the-art results using shells directly contacting a cyanobacterium (Supplementary Fig. 9) and around 1.5 times higher than those encapsulated in a disordered structure (Supplementary Fig. 11). Most importantly, our results confirm that the ordered yolk-shell encapsulated cyanobacteria

with uniformly organized nanoporosity not only maintain regular biological activity as native cells at low photon flux densities but may also exhibit significantly (up to 250% at 500 μE m^−2^ s^−1^) enhanced biological activity at higher photon flux densities (Fig. 4h). At excessively high photon flux densities, even in the presence of silica shell and void buffer, high light stress can induce some photoinhibition effect and lead to a slight decrease of oxygen production. However, compared to native cyanobacteria without any protection, the oxygen production rate of the cyanobacteria encapsulated in yolk-shell structure have a much higher oxygen production rate. We therefore suggest that the presence of interstitial voids in the yolk-shell encapsulation minimizes interfacial interactions with the cyanobacterial membrane as often observed in the conventional cell-contacting encapsulation strategy (Supplementary Fig. 9) or offer better permeability than disordered shells (Fig. 4h) for improved photosynthetic activity.

### Specific size-dependent permeability

Cyanobacteria encapsulated within the ordered yolk-shell structure were next evaluated for their viability upon exposure to two antibacterial agents: 10 nm silver nanoparticles and PDADMAC (polydiallyldimethylammonium chloride, molecular weight of 200 000 to 350 000 Da) and a lipophilic molecule: octane, as compared to native cyanobacteria and cyanobacteria encapsulated in disordered shells (Supplementary Fig. 11) with a highly irregular pore-size distribution ranging from 2 to 50 nm (Supplementary Fig. 11d), and in shells directly contacting the cells (Supplementary Fig. 9).

The encapsulation of cyanobacteria in an ordered yolk-shell structure leads to a very high viability of >90%, while due to the negative stresses resulting from the direct contact between the cell surface and the shell, the cell viability is reduced to ∼70%. The uniform pore-size distribution of the hierarchical ordering poses clear size-limitations to the permeability of the shells. Indeed, our yolk-shells with uniformly organized nanoporosity and ordered packing allow only size-selective permeability such as small inorganic salt molecules (Table 1 and Supplementary Fig. 13).

Upon supplementation of the growth medium with 10 nm diameter silver nanoparticles, the cyanobacteria encapsulated within ordered yolk-shells possessing 6.5 and 10.5 nm pores retained a very high viability (around 80%), while cyanobacteria in yolk-shells with a larger pore size (20.0 nm) had a lower viability (50%) (Table 1 and Supplementary Fig. 13). Native cyanobacteria and cyanobacteria encapsulated in disordered shells had statistically similar very low viabilities of less than 15% (Table 1 and Supplementary Fig. 13), demonstrating the inefficacy of the disordered shells in protecting the cyanobacteria against silver nanoparticles due to the broad distribution of shell pore sizes. The viability of the cells encapsulated in shells directly contacting a cell decreases to a low value of only around 35%, indicating again that the cell protection provided by the ordered yolk-shell structure is much more efficient than that provided by cell-contacting structures.

PDADMAC, with a molecular weight of 200 000 to 350 000 Da was unable to efficiently penetrate our ordered yolk-shells with uniform pores and kill cyanobacteria. Only less than 1% of native cyanobacteria survived after 1 day in PDADMAC supplemented medium, but encapsulation in ordered yolk-shells of different nanoporosities allowed over ∼75% of the cyanobacteria to survive. For comparison, the cyanobacteria encapsulated in cell-contacting cells show a viability of around 55%, while only 13% of the cyanobacteria survived when encapsulated by a disordered shell (Table 1 and Supplementary Fig. 13).

Although allowing penetration of small inorganic salt molecules, the hydrophilic silica nanoparticles forming the ordered outer shells in our capsules yielded highly effective protection against hydrophobic octane. 12% of native cyanobacteria, 39% of cyanobacteria encapsulated in disordered shells and only 1% of cyanobacteria encapsulated in cell-contacting shells were found viable after growth in medium supplemented with octane. On the other hand, our cyanobacteria with yolk-shell encapsulation yielded significantly higher viability of ∼80%, independent of the pore size. The shells offer protection against octane as the packing layer is composed of hydrophilic silica particles, which contain rich silicon hydroxyl (Si-OH) groups. We suggest that the hydroxyl groups, in combination with a high surface area and nutrient-filled interstitial voids, prevent the entry of hydrophobic organic molecules. The cyanobacteria encapsulated in disordered shells show a much lower survival ability, very likely due to the existence of too large pores that cannot completely prevent the penetration of the very small lipophilic octane molecules. The extremely low viability of the cyanobacteria encapsulated in cell-contacting shells could be attributed to the lack of the nutrient-filling interstitial voids, which can play a critical buffering role as the second barrier against the penetration of octane molecules.

Because of its specific size-dependent permeability and ordered yolk-shell biocompatible structure, the survival ability of encapsulated cyanobacteria against toxic chemical environments is significantly strengthened: around 5.9, 2.3 and 10-fold higher for 10 nm silver nanoparticles (an antimicrobial agent), 6, 1.4, 70 to 80-fold higher for PDADMAC (an antimicrobial molecule) and 2, 40 and 7-fold higher for octane than cells encapsulated in disordered shells, cells encapsulated in cell-contacting shells and native cyanobacteria, respectively.

In addition to toxic chemical environments, the yolk-shell structure can offer encapsulated cyanobacteria superior protection against harsh biological or physical conditions, such as resistance against lysozyme (Supplementary Fig. 17) and high temperature (Supplementary Fig. 18 and Table 2). Only about 50% of native cyanobacteria remain intact after exposure to lysozyme for 4 h, whereas this is 70% for the case of encapsulated cyanobacteria (Supplementary Fig. 14). It is worth noting that the cyanobacteria encapsulated in ordered yolk-shells were much less affected by lysozyme than those in disordered shells, suggesting that the shells with ordered porosity provide better cell protection.

The viability of the native, the cell-contacting shell encapsulated and the disordered shell encapsulated cyanobacteria after 1 h at 50°C is below 35%, while that of all yolk-shell encapsulated cells is above 50% (Supplementary Fig. 18 and Table 2). We suggest that the nutrient-filling interstitial voids in yolk-shell structure serve as a thermal-protective layer to slow down the heat transfer from the environment to cells, thus enhancing the survivability of encapsulated cyanobacteria at high temperature.

The yolk-shell structure of the single-cell capsules also shows high stability in the culture condition. Three grouped cyanobacteria encapsulated in the yolk-shells formed by different sized silica colloids were cultured in the medium for 5 days and subsequently characterized using SEM. The SEM micrographs (Supplementary Fig. 19) show that the shell of most of the encapsulated cyanobacteria is still intact and uniform. This indicates that the yolk-shell structure is maintained after the 5-day culture, thus evidencing the high stability of the yolk-shell structure.

### Specific molecular recognition abilities

The hydrophilic silica nanoparticles forming the ordered porous shell containing a high number of Si-OH groups may offer further possibilities for appropriate chemical modification of the shells for molecular recognition abilities.

**Figure 5.**
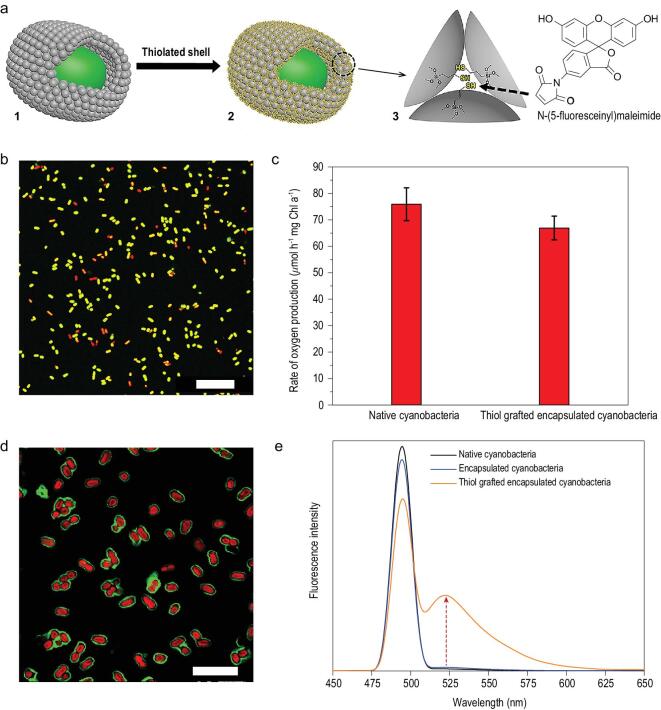
Molecular recognition of thiolated yolk-shell single-cell capsules. (a) Schematic illustration of the functionalization of a yolk-shell around cyanobacteria by thiol-grafting to create molecular recognition ability for a maleimide derivative: (1) encapsulated cyanobacterium, (2) thiolated silica nanoparticles in a yolk-shell around a cyanobacterium, (3) voids between thiol-grafted silica nanoparticles in the colloidal packing layer of yolk-shell encapsulated cyanobacterium. (b) Merged CLSM micrograph of FDA stained cyanobacteria in a yolk-shell with thiol-grafted silica nanoparticles as the packing layer showing above 90% viability. Scale bar: 25 μm. (c) Rate of oxygen production of native cyanobacteria and thiol-grafted yolk-shell encapsulated cyanobacteria at a photon flux density of 200 μE m^−2^ s^−1^. Error bars denote standard deviation over three experiments with separately cultured cyanobacteria. (d) Merged CLSM micrograph of thiolated yolk-shell encapsulated cyanobacteria with pore size of 10.9 nm, showing red fluorescent bacteria and green fluorescent maleimide molecules coupled to thiol grafted nanoparticles. Scale bar: 10 μm. (e) Fluorescence spectra of native cyanobacteria and yolk-shell encapsulated cyanobacteria with and without grafted thiol, confirming the presence of maleimide on the thiolated shells, as evidenced by the fluorescence peak at 523 nm indicated by the arrow.

To demonstrate this possibility, we covalently grafted thiol groups to the silica nanoparticles in the colloidal packing layer of encapsulated cyanobacteria with (3-mercaptopropyl)trimethoxysilane (MPTMS) in dry dodecane to enable specific molecular recognition of maleimide derivatives (Fig. 5a). The thiol-grafted yolk-shells had a high viability (Fig. 5b) and a similar rate of oxygen production as native cyanobacteria (Fig. 5c), showing again the high survival ability against lipophilic molecules such as dodecane. After exposure of thiol grafted single-cell capsules to a maleimide solution for 1 h, maleimide coupling to the shells generates a green fluorescent ring around individual encapsulated, red fluorescent cyanobacteria (Fig. 5d). The fluorescence spectra of cyanobacteria encapsulated in thiol-grafted yolk-shells showed a peak at 523 nm, characteristic of maleimide, that was absent for native cyanobacteria and cyanobacteria encapsulated in non-functionalized yolk-shells (Fig. 5e). This demonstration shows the possibilities in the incorporation of functional groups in our single-cell encapsulation strategy, enabling specific molecular recognition abilities and potentially, a wide range of other functionalities.

## CONCLUSION

We have developed a strategy to synthesize yolk-shell encapsulation for single cells. This is achieved through self-assembly of protamine on the surface of cyanobacteria on to which colloidal silica nanoparticles are subsequently organized. Our experimental evidence on the internalization of protamine into the cell cytoplasm and its kinetics indicate that the process is quick and spontaneous. The precisely- and time-controlled internalization of the protamine is a crucial step and is exploited to create an interstitial void between the cell surface and the ordered silica colloids packing shell with uniformly organized nanoporosity in the encapsulated cyanobacterium. This void offers buffering possibilities and a biocompatible environment to the cyanobacterium to survive and grow through a facile exchange of nutrients and waste products through the ordered colloidal packing layer. To underscore the importance of protamine internalization, we also demonstrate that PEI (a positively charged molecule like protamine) can be internalized into the cell wall-removed microalgae cells, but cannot be internalized by the cyanobacteria and therefore, can only be used to induce ordered assembly of silica nanoparticles to create cell-contacting shells around the cell surface of cyanobacteria. The rate of photosynthetic oxygen production of our yolk-shell encapsulated cyanobacteria at a large range of photon flux densities (0–500 μE m^−2^ s^−1^) is 3 to 4-fold higher than the state-of-the-art results using cell-contacting shell encapsulated cyanobacteria. At the same time, strong resistance to harsh environments of the yolk-shell encapsulated cyanobacteria is ensured by specific size-dependent permeability and the biocompatible, buffering environment in the voids. The survival ability of the encapsulated cells against silver nanoparticle, PDADMAC antimicrobials and octane-rich solution is significantly strengthened: at least around 5.9, 2.3 and 10-fold for 10 nm silver nanoparticles, 6, 1.4 and 70 to 80-fold higher for PDADMAC and 2, 40 and 7-fold higher for octane than the cells encapsulated by disordered shells, cells encapsulated by cell-contacting shells and native cyanobacteria, respectively. In addition, our ordered yolk-shell structure enables us to offer encapsulated cyanobacteria with superior protection against harsh biological or physical conditions, such as resistance against lysozyme or high temperature. Our yolk-shell structure can also be equipped with molecular recognition abilities. Compared with currently existing encapsulation methods leading to shells that directly contact a bacterium, our ordered yolk-shell design strategy is far superior in maintaining biological activity and stability in a harsh environment. Our experimental demonstration and study of protamine internalization and its kinetics could potentially be further used in the field of biotechnology. It is envisioned that single cells encapsulated in our ordered yolk-shell structures have a broad scope in a wide range of applications with specific functionalities, including in photobioreactors, biochips, biosensors, biocatalysis, biofuel reactors and controlled delivery therapeutics.

## METHODS

### Cyanobacterial culturing and harvesting

*Synechocystis sp. PCC 7002* were cultured at 22°C [63] in 1:1 BG-11: ASN supplemented with 1 μg dm^−3^ vitamin B_12_ under continuous illumination using a fluorescent strip light in a shaking incubator. Cyanobacteria were cultured for 5 days. The growth was terminated when photosynthetic activity was at its maximum. Cyanobacteria were harvested by centrifugation (3620 *g* for 5 min) at 4°C. Please note that the centrifugation (3620 *g* for 5 min) at either 4°C or 22°C will not influence the cell activity of cyanobacteria (Supplementary Fig. 20). When experiments required, the encapsulated cyanobacteria were either suspended in a fresh growth medium, with or without specific antimicrobial supplement or in 0.1 M phosphate buffered saline at pH 7.4 (PBS) to an optical density OD_730 nm_ of 1.3. The relation between OD_730 nm_ versus bacterial numbers is presented in Supplementary Fig. 21.

### Encapsulation procedure

For encapsulation, cyanobacteria in suspension were supplemented with protamine sulfate (Sigma-Aldrich, USA) to a concentration of 0.1 mg mL^−1^. After 5 min priming with protamine, cyanobacteria were washed with PBS and mixed with silica colloidal nanoparticles (1.0 mg mL^−1^, LUDOX^®^, Sigma-Aldrich) for 20 min, after which cyanobacteria were rewashed with PBS to yield an ordered yolk-shell (Fig. 1a). To create colloidal packing layers with different porosity, silica nanoparticles with different diameters were used (LUDOX^®^ SM-30, LUDOX^®^ HS-40, LUDOX^®^ TMA, Sigma-Aldrich, USA).

Advantages of ordered yolk-shell encapsulation of cyanobacteria were compared with those of cell-contacting and disordered shells. Cell-contacting shells were prepared by priming the cyanobacteria in PBS containing 0.1 mg mL^−1^ polyethylenimine (PEI, MW∼25 000, Polysciences, Inc., USA) for 5 min, followed by washing in PBS and mixing with 14.9 nm diameter silica colloidal nanoparticles (1.0 mg mL^−1^, LUDOX^®^ HS-40, Sigma-Aldrich, USA). The thickness of the cell-contacting shell can be increased with the repeated PEI and silica nanoparticle deposition. Disordered shells were prepared by priming the cyanobacteria in PBS containing 0.1 mg mL^−1^ protamine sulfate (Sigma-Aldrich, USA) for 5 min, followed by washing in PBS and mixing with a 0.15 M silicic acid solution.

The cytotoxicity of protamine on cyanobacteria was determined by the viability of the cyanobacteria incubated in PBS containing protamine sulfate with different concentrations (0.1, 0.2, 0.5 and 1.0 mg mL^−1^). The viability of cyanobacteria was measured using fluorescein diacetate (FDA, Sigma-Aldrich, USA) staining and the protocol is described in detail in the below section (Monitoring of bacterial viability, growth and oxygen production).

### Characterization of encapsulating shells

SEM and EDX analysis on the encapsulating shells were performed using a JEOL 7550F (JEOL, Japan), operated at 15 kV. For specimen preparation, encapsulated cyanobacteria were dehydrated in a series of solutions with increasing percentages of ethanol and supercritically dried in a Leica EM CDP030 (Leica, Germany). HAADF-STEM was carried out on an FEI Tecnai G2 microscope, operated at 200 kV, with specimen preparation as described above. TEM was conducted using TECNAI10 (TECNAI, Japan), operated at 80 kV. Specimens for TEM were prepared by fixing encapsulated cyanobacteria with gluteraldehyde, OsO_4_, followed by dehydration in acetone and embedding Epon 812/Araldite M resin. Subsequently, thin sections were cut using an ULTRACUT UCT ultramicrotome (Leica, Germany). CLSM images were acquired using a Leica TCS SP5 (Leica, Germany). The encapsulating shells were fluorescence-labelled by the protocol of pHrodo^TM^ Green (Thermo Fisher Scientific, USA). First, a pHrodo^TM^ Green stock solution (0.1% v/v) was prepared by adding the fluorescence label into 4-(2-hydroxyethyl)-1-piperazineethanesulfonic acid (HEPES, Sigma-Aldrich, USA) buffer (0.05 M, pH 7.4). For experiments, a 1 mL aliquot of the stock solution was added into the encapsulated cyanobacteria suspension and incubated for 30 min at ambient temperature under shaking and subsequently examined using CLSM. To present the cell-in-shell structure more clearly, CLSM micrographs captured in green fluorescent channel and the corresponding ones captured in red fluorescent channel were overlapped to merge two-colour micrographs. Zeta potentials of native cyanobacteria and encapsulated ones with OD_730 nm_ of 0.2 were measured in 0.01 M PBS at pH 7.4, using a Horiba SZ 100 nanoparticle analyser (Horiba, Japan).

### Investigation of internalization behaviour

A fluorescent dye (pHrodo^TM^ iFL Green STP Ester (amino reactive), Thermo Fisher Scientific, USA), which enables us to distinguish the intracellular environment by its pH-dependent fluorescent responses was used to label the protamine and PEI. The labelling procedures followed the protocol of pHrodo^TM^ iFL Green STP Ester (amino reactive) (Thermo Fisher Scientific, USA). First, protamine sulfate (Sigma-Aldrich, USA) or PEI (MW∼25 000, Polysciences, Inc., USA) was dissolved in a PBS supplemented with 0.1 M NaHCO_3_. The final concentration of protamine or PEI was 0.2 mg mL^−1^. 500 μL protamine or PEI solution was mixed with 100 μL pHrodo^TM^ iFL dye stock solution (2 mg mL^−1^). For the labelling reaction, the mixture was incubated for 50 min at room temperature and was protected from light. The obtained labelled protamine or PEI was further treated using dialysis for purification. To track the internalization behaviour of cyanobacteria (or cell wall-removed microalgae cells (*Chlamydomonas reinhardtii*)), a cyanobacterial (or cell wall-removed microalgae cells) suspension supplemented with the labelled protamine or PEI (0.1 mg mL^−1^) was monitored as a function of time using fluorescence spectroscopy Perkin-Elmer LS 45 luminescence spectrometer (PerkinElmer, USA).

A green fluorescent molecule (5(6)-carboxy-fluorescein *N*-hydroxysuccinimide ester, Sigma-Aldrich, USA) was used to label the protamine and PEI to track the internalization behaviours of cyanobacteria. To synthesize fluorescent protamines, 1.0 mg of the 5(6)-carboxyfluorescein *N*-hydroxysuccinimide ester in 200 μL of dimethyl sulfoxide was added to 10 mg of protamine sulfate (Sigma-Aldrich, USA) or PEI (MW∼25 000, Polysciences, Inc., USA) in 2000 μL of PBS, and allowed to react overnight at 4°C. The obtained labelled protamine and PEI were further treated using dialysis for purification. To track the internalization behaviour of cyanobacteria, a cyanobacterial suspension supplemented with the labelled protamine or PEI (0.1 mg mL^−1^) was monitored as a function of time using CLSM Leica TCS SP5 (Leica, Germany).

### Monitoring of cyanobacterial viability, growth and oxygen production

The viability of cyanobacteria before and after encapsulation was measured using fluorescein diacetate (FDA, Sigma-Aldrich, USA) staining. An FDA stock solution (10 mg mL^−1^) was prepared by dissolving FDA in acetone. For experiments, a 5 μL aliquot of the stock solution was mixed with 1 mL of a cyanobacterial suspension. The mixture was incubated for 30 min at ambient temperature under shaking and subsequently examined using CLSM. To present the viability clearly, CLSM micrographs acquired in green fluorescent channel and the corresponding ones acquired in red fluorescent channel were overlapped to merge two-colour micrographs. Growth kinetics were evaluated by suspending cyanobacteria with or without encapsulation in medium (1:1 BG-11: ASN-III supplemented with 1 μg dm^−3^ vitamin B_12_) to an OD_730 nm_ of 0.2 and monitoring growth for 13 days, with aliquots taken at regular times for optical density measurements. Photosynthetic production of oxygen by native and encapsulated cyanobacteria was measured at 20°C by monitoring oxygen production with a Clark-type oxygen electrode (Hansatech Instrument, Kings Lynn, UK). The rate of photosynthetic oxygen production was measured on 1 mL cyanobacterial suspensions in presence of 10 mM NaHCO_3_. The *Chlorophyll a* in cyanobacteria was extracted by cooled methanol for 20 min. The absorbance of the extracted *Chlorophyll a* suspension at 665 nm (A_665_) and 720 nm (A_720_) was measured using Lambda 35 UV/VIS spectrometer (PerkinElmer, USA). The concentration of *Chlorophyll a* in cyanobacteria is calculated according to an equation: *Chl a* (μg mL^−1^) = 12.9447 (A_665_ – A_720_). Photosynthetic carbon fixation was characterized by using ^14^C labelled NaHCO_3_ transfer from the medium into the cyanobacteria upon growth for 1 h and 24 h. After growth, suspensions were centrifuged (3 620 *g* for 5 min) at 4°C and the cyanobacterial pellet mixed with an Insta-Gel Packard (PerkinElmer, USA) scintillation cocktail by vortexing. Radioactivity was measured using a Beckman scintillation counter LS 6000 SC (Beckman Coulter, USA) and the standard external method for quench correction.

### Yolk-shell protection of cyanobacteria against silver nanoparticles, PDADMAC, an octane-rich environment, lysozyme and high temperature

To demonstrate protection against silver nanoparticles, 50 μL suspensions of 10 nm diameter silver nanoparticles (Sigma-Aldrich, USA) (0.02 mg mL^−1^) were added to 1 mL suspensions of encapsulated cyanobacteria (OD_730 nm_ 1.3). Cyanobacteria were encapsulated in ordered yolk-shells with a colloidal packing layer composed of silica nanoparticles with diameters of 9.2, 14.9 or 31.0 nm or a disordered shell or a cell-contacting shell as a control. After 12 h of growth, viability was assessed using FDA (Sigma-Aldrich, USA) staining and CLSM (Leica, Germany). In a similar protocol, shell protection against a toxic macromolecule polydimethylammonium chloride (PDADMAC, MW ∼ 200 000–350 000 Da, Sigma-Aldrich, USA) was evaluated. 2 μl of an aqueous 20 wt% solution of PDADMAC was added to 2 mL growth medium with native and encapsulated cyanobacteria (OD_730 nm_ 1.3) and allowed to grow for 24 h after which viability was assessed (see above). Protection against a hydrophobic environment was evaluated by adding 100 μL of octane (Sigma-Aldrich, USA) to 2 mL of growth medium with native and encapsulated cyanobacteria (OD_730 nm_ 1.3) and measuring viability after 24 h, as described above. Shell protection against lysozyme was evaluated by calculating the cell disintegration of native and encapsulated cyanobacteria affected by lysozyme. Native and encapsulated cyanobacteria were pre-treated at −20°C for 1 h, in order to destabilize the cell wall to facilitate the observation of cell disintegration (please note that the pre-treatment at −20°C for 1 h will not cause any cell disintegration [64]). Lysozyme (from chicken egg white, protein ≥90%, ≥40 000 units mg protein^−1^, 14.3 kDa, Sigma-Aldrich, USA) was added into 1 mL suspensions of native and encapsulated cyanobacteria (OD_730 nm_ 1.3) with a final concentration of 1 mg mL^−1^. The cyanobacteria-lysozyme mixture was placed at 37°C. The ratio of cell disintegration was followed over time by calculating the percentage of the real-time OD_730 nm_ to that prior to the lysozyme treatment. Finally, shell protection against high temperature was evaluated by measuring the viability of native and encapsulated cyanobacteria (following the protocol for viability assessment described above) after incubation at 50°C for 1 h.

### Incorporation of functional groups in the ordered yolk-shell with specific molecular recognition abilities

After encapsulation, the cyanobacteria (OD_730 nm_ 1.3) were harvested and suspended into 5 mL dry dodecane (Sigma-Aldrich, USA) after which 50 μL (3-mercaptopropyl)trimethoxysilane (MPTMS, Sigma-Aldrich, USA) was added. Next, the suspension was placed in a shaking incubator for 10 min to covalently graft the thiol to the silica nanoparticles and encapsulated cyanobacteria were washed twice with PBS and added into a solution of *N*-(5-fluoresceinyl)maleimide (Sigma-Aldrich, USA) for 1 h, prepared by filtering an aqueous solution of *N*-(5-fluoresceinyl)maleimide (1 mg mL^−1^, Sigma-Aldrich, USA) and mixing the resulting solution to PBS with 1:1 (v/v) ratio. The maleimide interaction with thiol-grafted encapsulated cyanobacteria was demonstrated using CLSM (Leica TCS SP5, Germany) and fluorescence spectroscopy Perkin-Elmer LS 45 luminescence spectrometer (PerkinElmer, USA). To make the interaction visible, CLSM micrographs captured in green fluorescent channel and the corresponding ones captured in red fluorescent channel were overlapped to merge two-colour micrographs.

## Supplementary Material

nwaa097_Supplemental_File
