# Supplementary material for: Single-cell yolk-shell nanoencapsulation for long-term viability with size-dependent permeability and molecular recognition
Source: Natl Sci Rev. 2020 May 9;8(4):nwaa097. doi: 10.1093/nsr/nwaa097 (PMC8288456; doi:10.1093/nsr/nwaa097)
Supplement: nwaa097_Supplemental_File [file nwaa097_supplemental_file.docx]

Supplementary Information

**Single-Cell Yolk-Shell Nanoencapsulation for Long-Term Viability with Size-Dependent Permeability and Molecular Recognition**

Li Wang^1,2^, Yu Li^1^, Xiao-Yu Yang^1★^, Bo-Bo Zhang^2^, Nöelle Ninane^3^, Henk J. Busscher^4^, Zhi-Yi Hu^1,5^, Cyrille Delneuville^2^, Nan Jiang^1,6^, Hao Xie^1^, Gustaaf Van Tendeloo^5, 7^, Tawfique Hasan^8^, and Bao-Lian Su^1,2★^


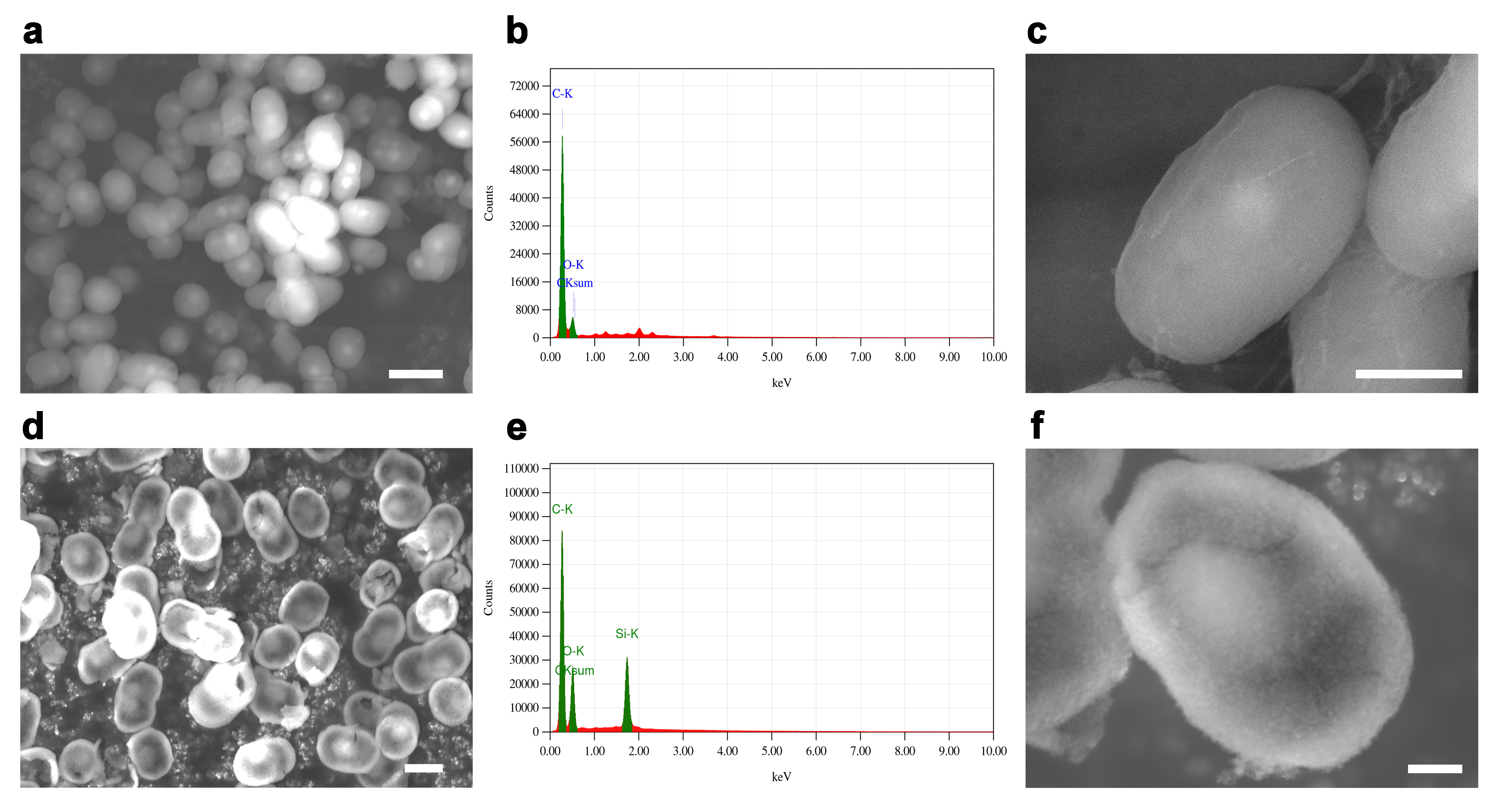


**Supplementary Figure 1. Morphology and chemical composition of cyanobacteria.** (**a,b**) SEM micrograph (**a**) and the corresponding EDX spectra (**b**) of native cyanobacteria. Scale bar, 2 μm. (**c**) SEM micrograph of a native cyanobacterium. Scale bar, 500 nm. (**d,e**) SEM micrograph, Scale bar, 2 μm. (**d**) and the corresponding EDX spectra (**e**) of cyanobacteria encapsulated in a protamine-assisted, hydrophilic silica, colloidal packing. (f) SEM micrograph of an encapsulated cyanobacterium in yolk-shell structure. Scale bar, 500 nm.


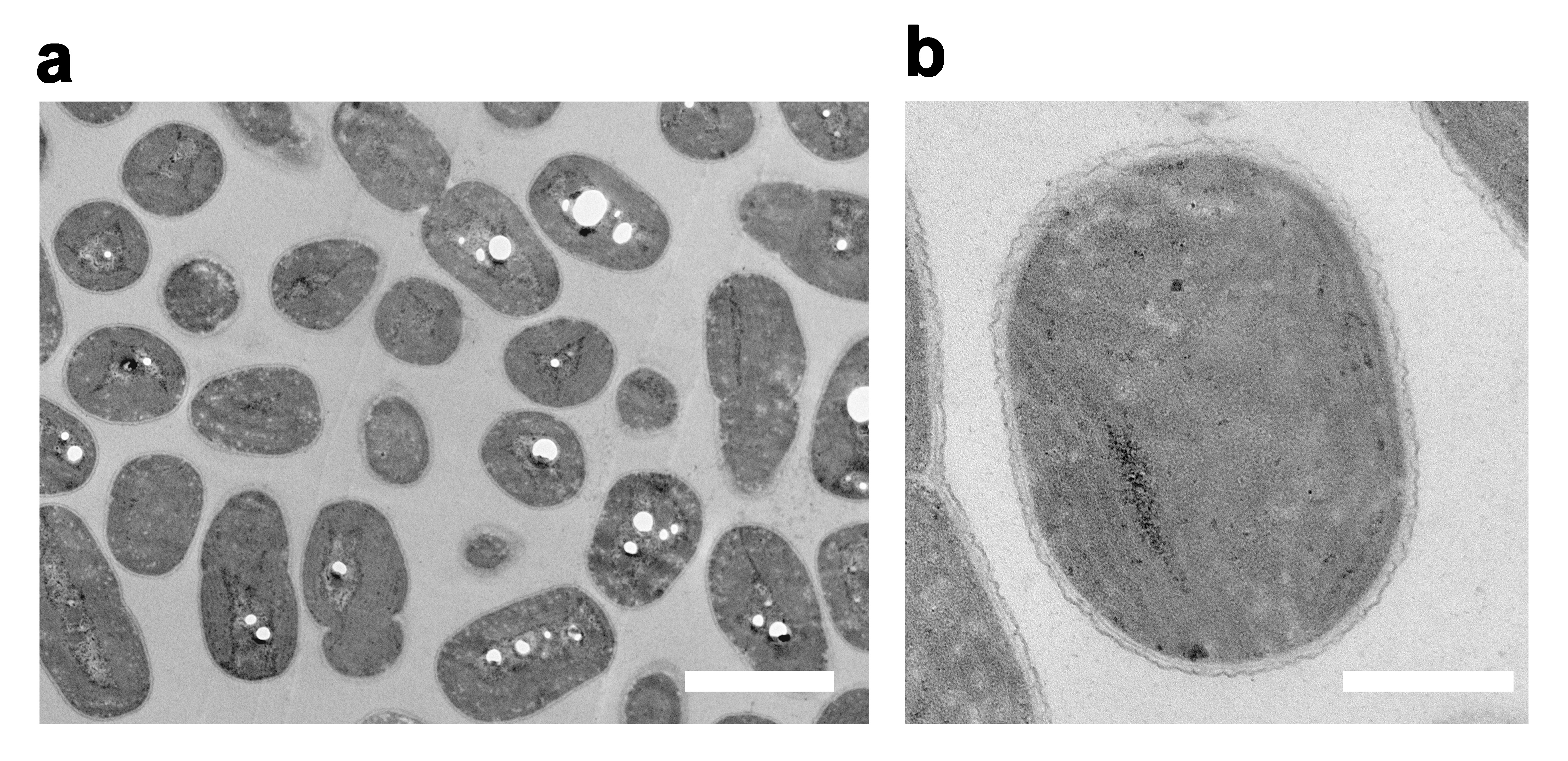


**Supplementary Figure 2.** TEM micrographs of (a) native cyanobacteria; scale bar, 2 μm and (b) a native cyanobacterium; scale bar, 500 nm.


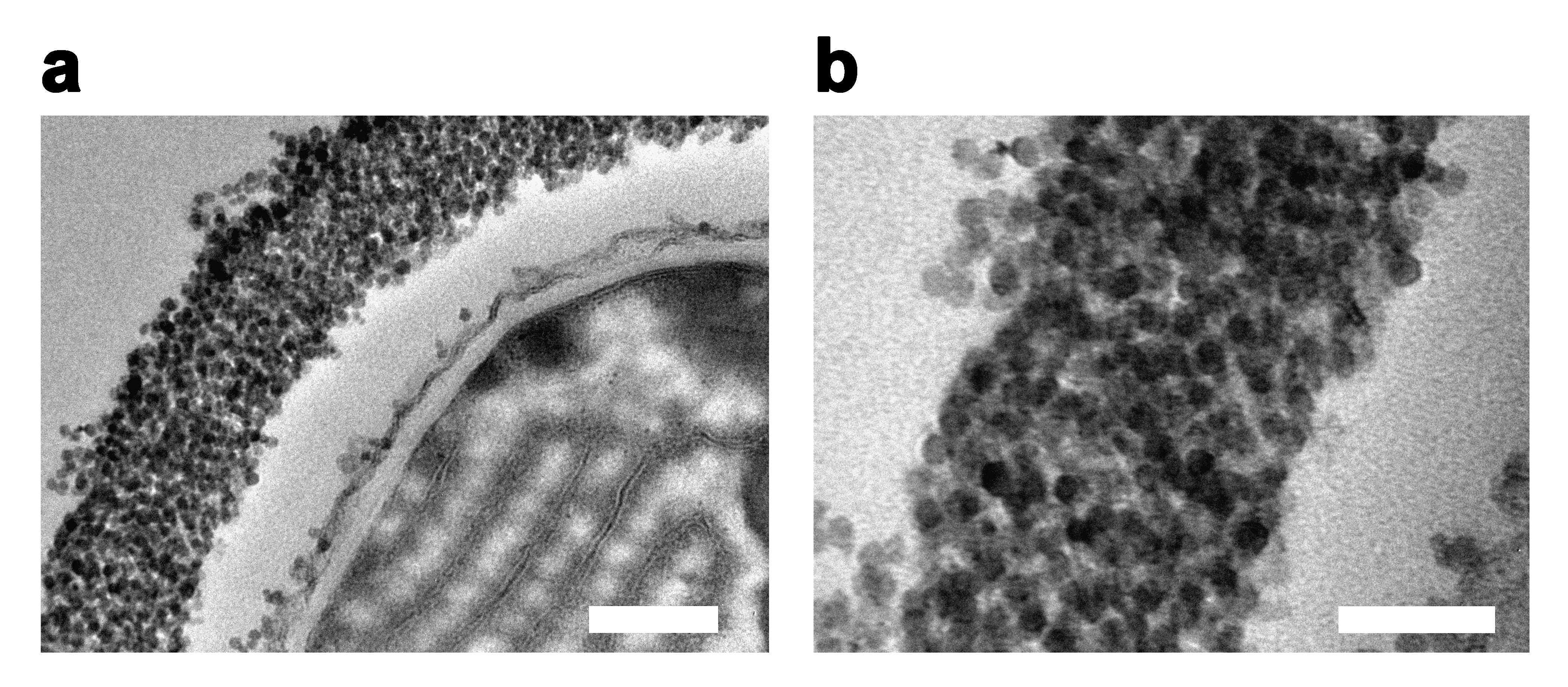


**Supplementary Figure 3. Structure of the silica shell and the cytoplasm of a single cell yolk-shell capsule.** (**a**) TEM micrograph of the silica shell and the cytoplasm of a single cyanobacterium capsule by protamine-assisted colloidal packing with nanoparticles of which diameter is 14.9 nm. Scale bar, 200 nm. (**b**) TEM micrograph of the silica shell formed by protamine-assisted colloidal packing with nanoparticles of which diameter is 14.9 nm. Scale bar 100 nm.


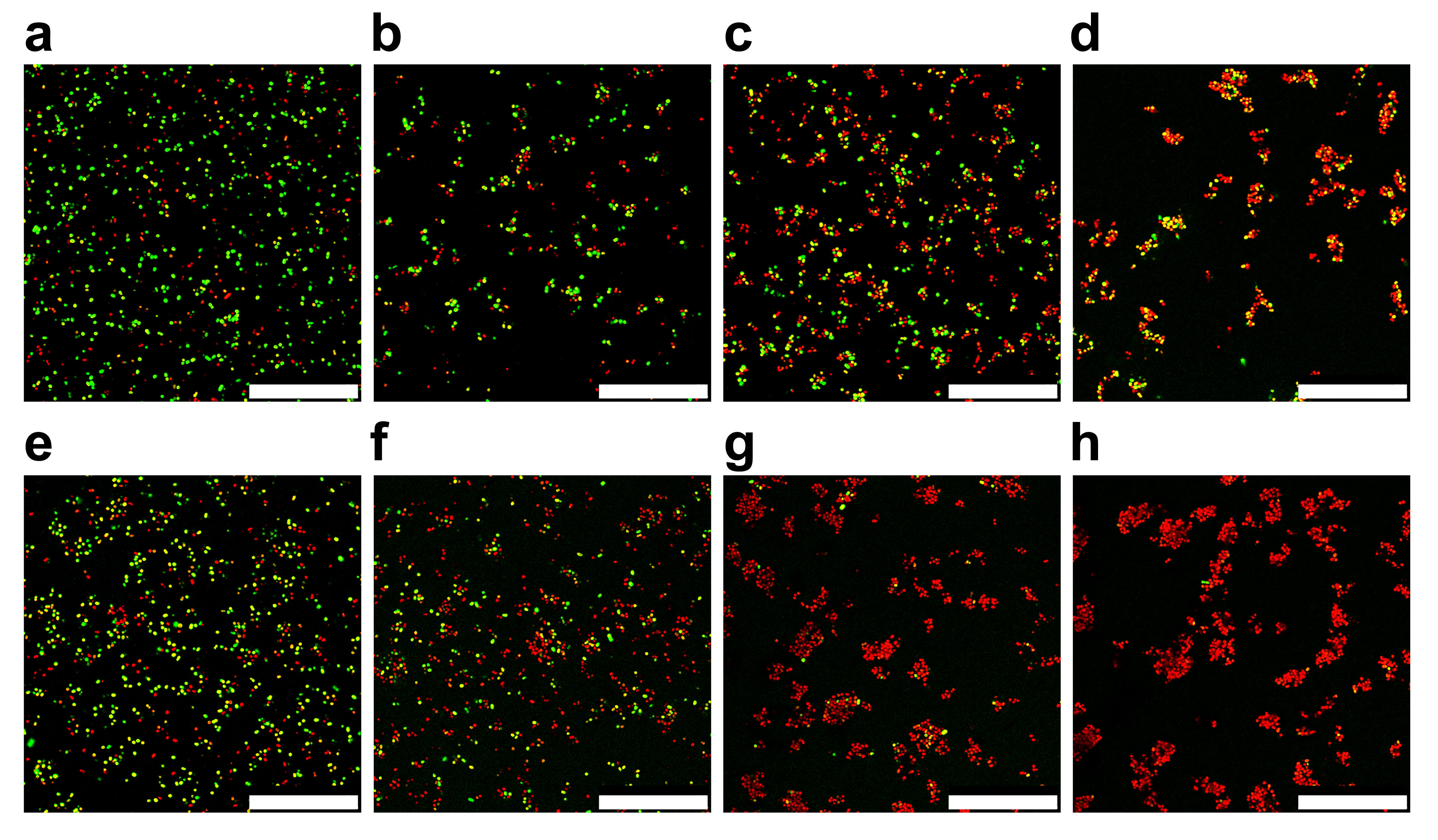


**Supplementary Figure 4. Cytotoxicity of protamine and PEI on cyanobacteria.** (**a-d**) Merged CLSM micrographs of FDA-stained cyanobacteria primed by protamine with the concentration of (**a**) 0.1, (**b**) 0.2, (**c**) 0.5 and (**d**) 1.0 mg mL^-1^. (**e-h**) Merged CLSM micrographs of FDA-stained cyanobacteria primed by PEI with the concentration of (**e**) 0.1, (**f**) 0.2, (**g**) 0.5 and (**h**) 1.0 mg mL^-1^. Greenish-yellowish fluorescent cyanobacteria are alive, while red fluorescent ones are dead. Scale bars, 50 μm.


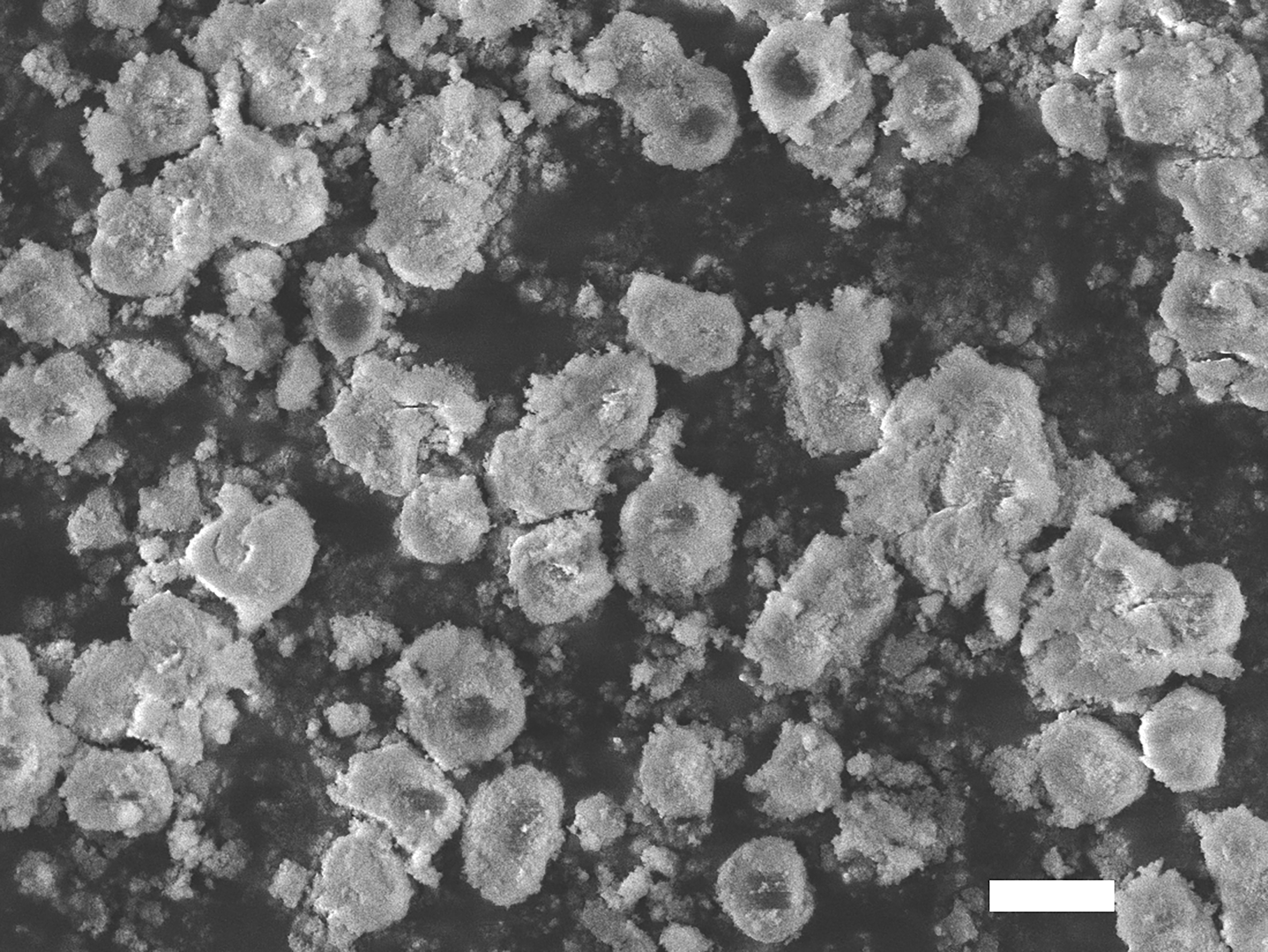


**Supplementary Figure 5. Influence of the low concentration of protamine on single cell encapsulation.** SEM micrograph of the single cyanobacterium capsules formed with the protamine concentration of 0.05 mg mL^-1^. Scale bar, 2 μm.


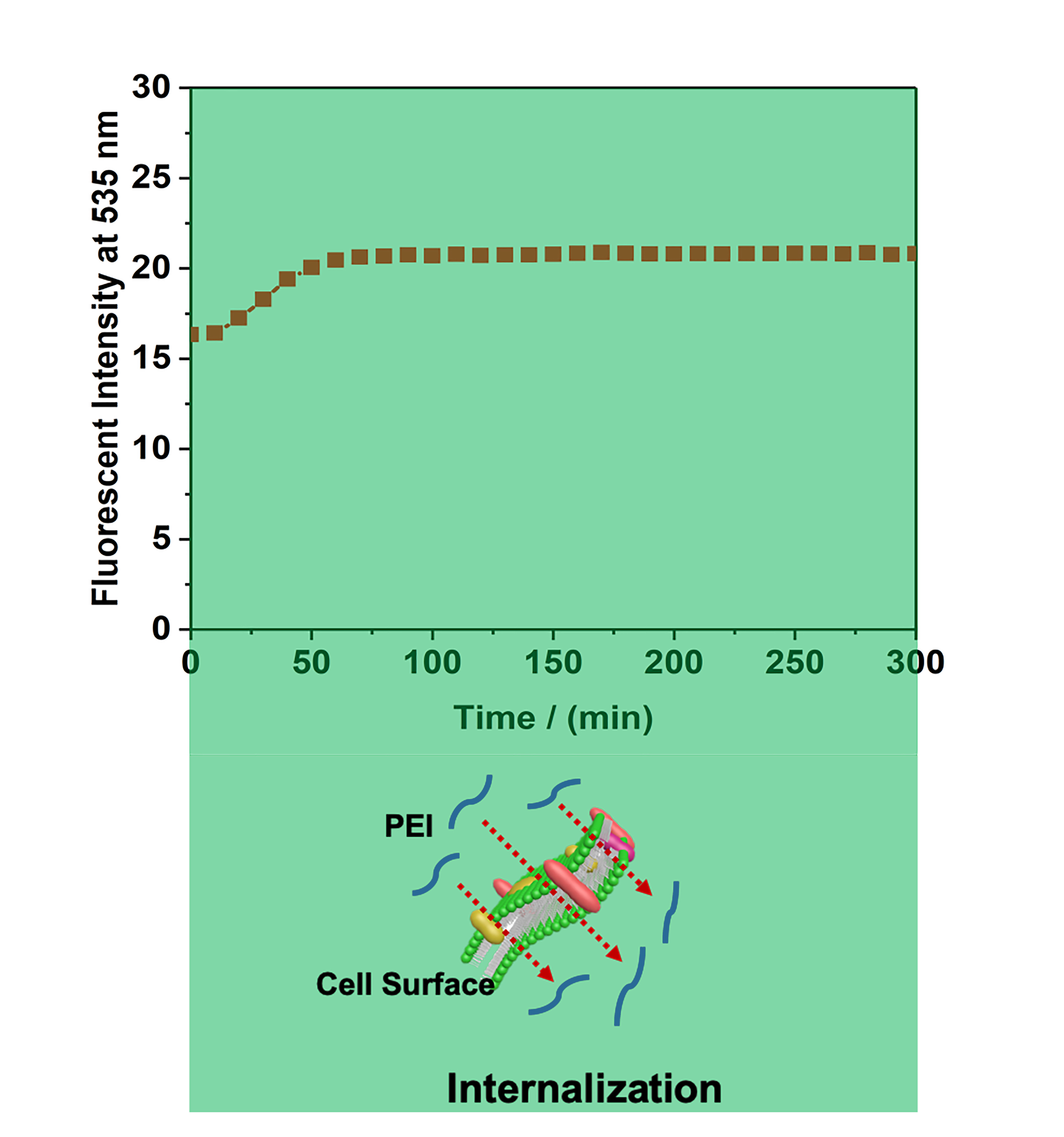


**Supplementary Figure 6. PEI Internalization behavior of cell-wall removed microalgae cells.** Fluorescence intensity at 535 nm of the cell wall-removed microalgae cells-PEI mixture with time obtained by an *in-situ* fluorometry spectroscopic study. The increase in fluorescent intensity represents the internalization of PEI into the cytoplasm of microalgae cells.


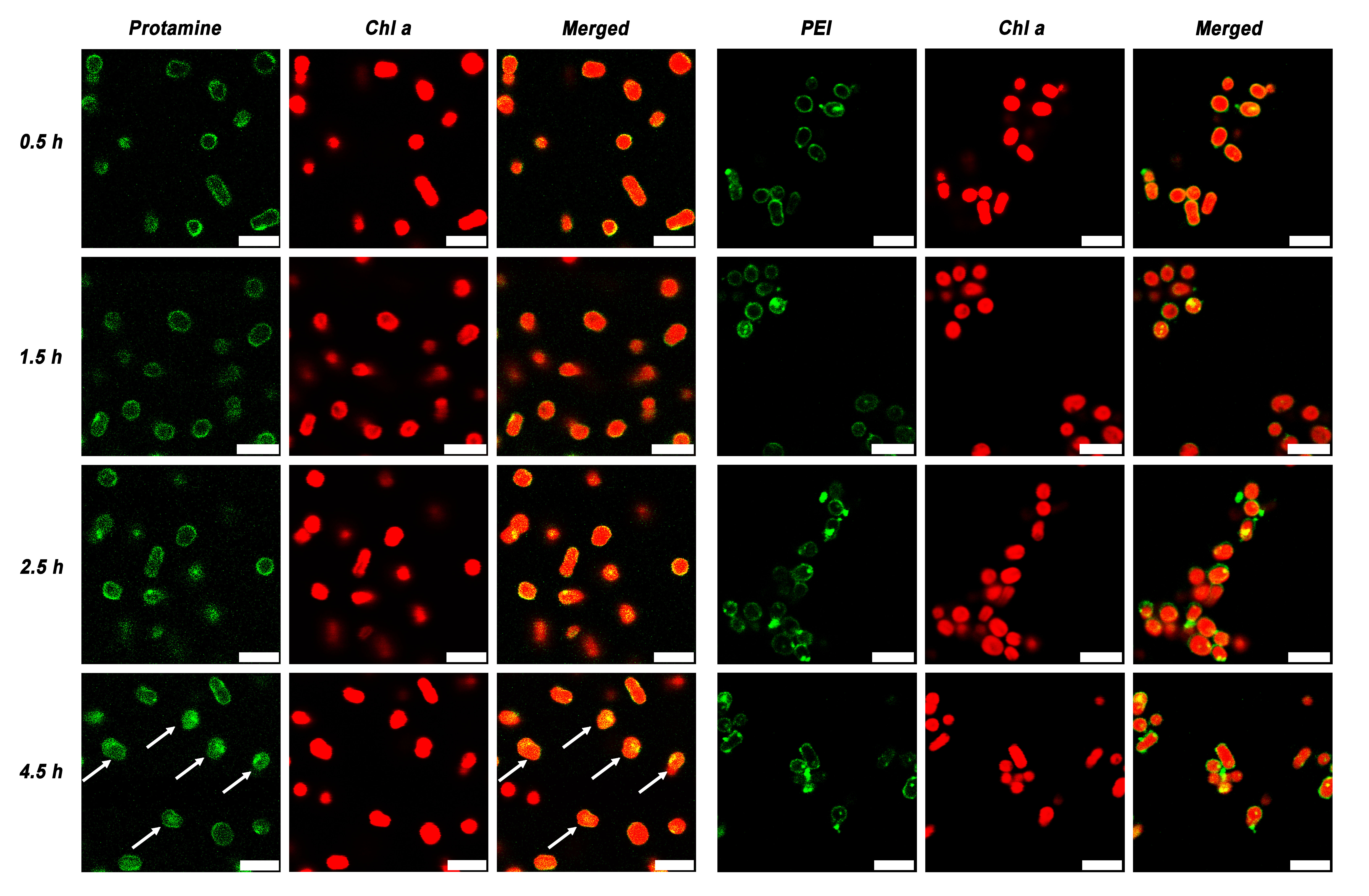


**Supplementary Figure 7. Internalization behavior of cyanobacteria.** CLSM micrographs of the cyanobacteria-protamine mixture and cyanobacteria-PEI mixture over time. Protamine and PEI were labelled with green fluorescent (6)-Carboxyfluorescein *N*-hydroxysuccinimide ester. Cyanobacteria containing *chorolophyll a* were indicated as red fluorescent spots. The cyanobacteria with internalized protamine after 4.5-h incubation were indicated by white arrows. Scale bars, 5 μm.


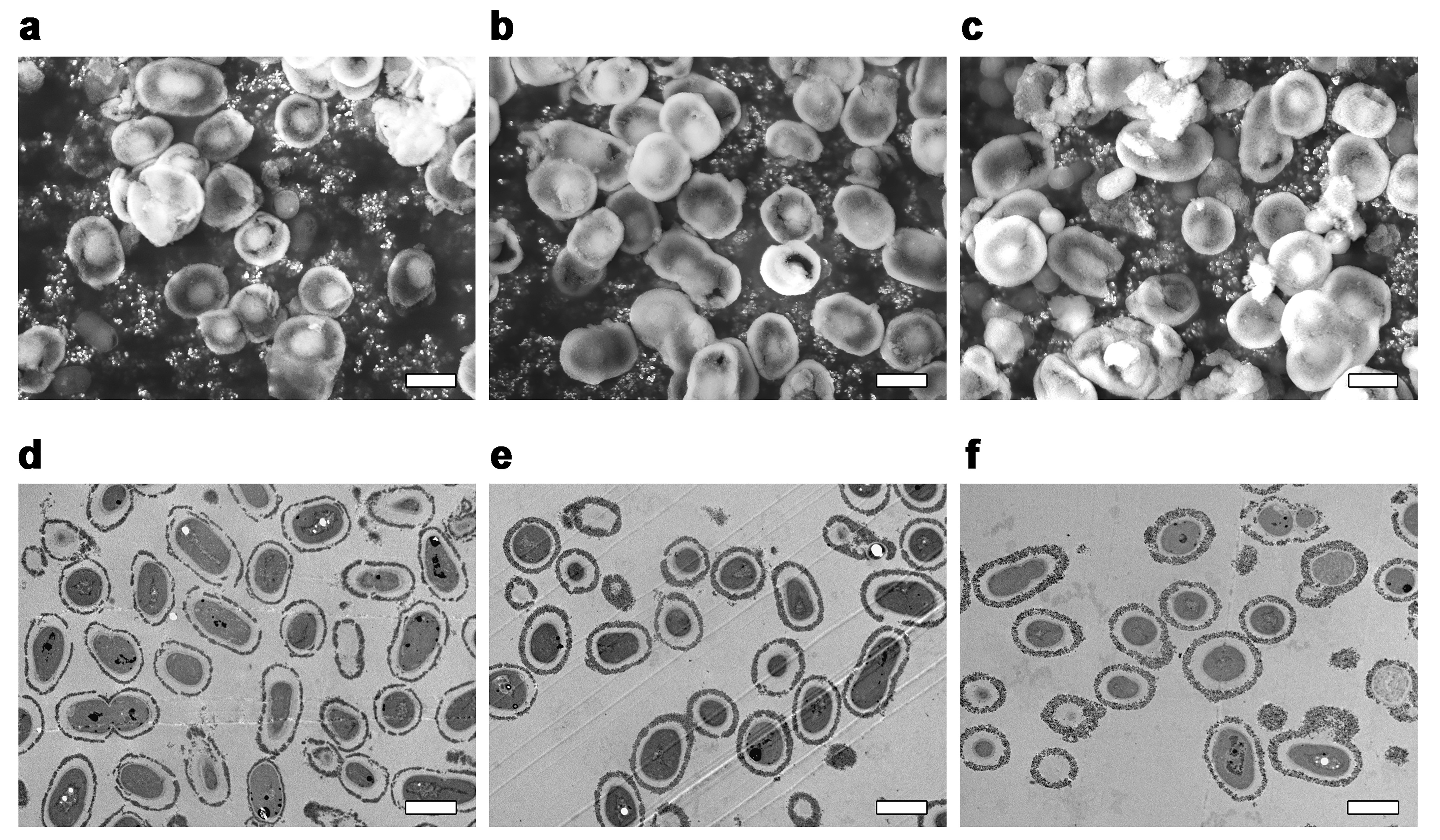


**Supplementary Figure 8. Morphology and structure of yolk-shell single cell capsules with different pore-sized shells.** (**a-c**) SEM micrographs of single cyanobacterium capsules by protamine-assisted colloidal packing with nanoparticles of different diameters of 9.2 (**a**), 14.9 (**b**) and 31.0 nm (**c**). Scale bars, 2 μm. (**d-f**) TEM micrographs of single cyanobacterium capsules by protamine-assisted colloidal packing with nanoparticles of different diameters of 9.2 (**d**), 14.9 (**e**) and 31.0 nm (**f**). Scale bars, 2 μm.


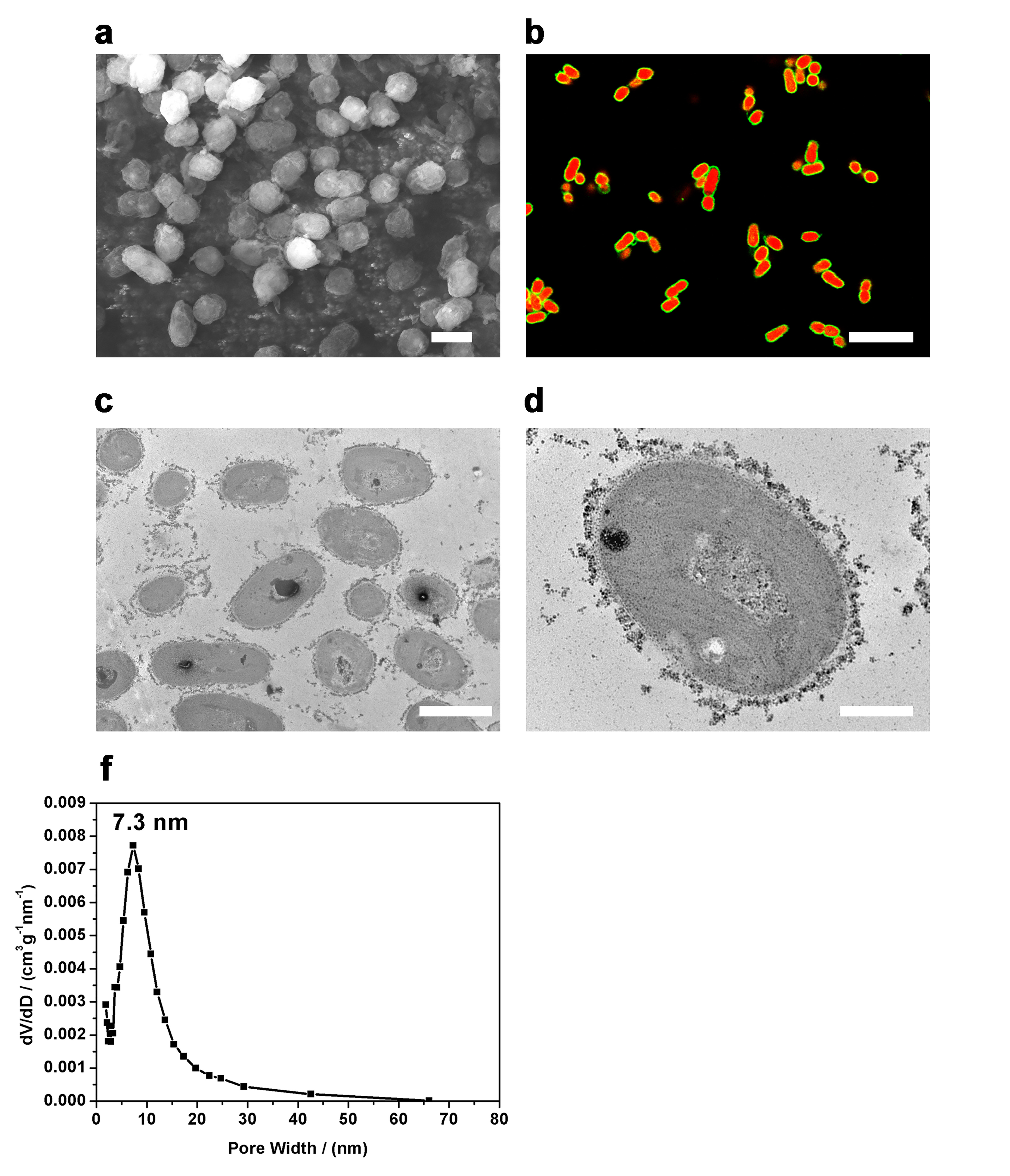


**Supplementary Figure 9. Characteristics of cyanobacteria encapsulated in cell-contacting shells.** (**a**) SEM micrograph of cyanobacteria encapsulated in cell-contacting shells. Scale bar, 2 μm. (**b**) CLSM micrograph of encapsulated cyanobacteria in cell-contacting shells, showing singly-encapsulated, red-fluorescent cyanobacteria and green-fluorescent silica shells without interstitial voids in between the bacteria and the shells. Scale bar, 10 μm. (**c**) TEM micrograph of cyanobacteria encapsulated in cell-contacting shells. Scale bar, 2 μm. (**d**) TEM micrograph of a cyanobacteria encapsulated in a cell-contacting shell. Scale bar, 500 nm. (**f**) Pore size distribution, peaking at 7.3 nm, of the cell-contacting shells employed for comparison, determined using BJH analysis of nitrogen adsorption-desorption isotherms.


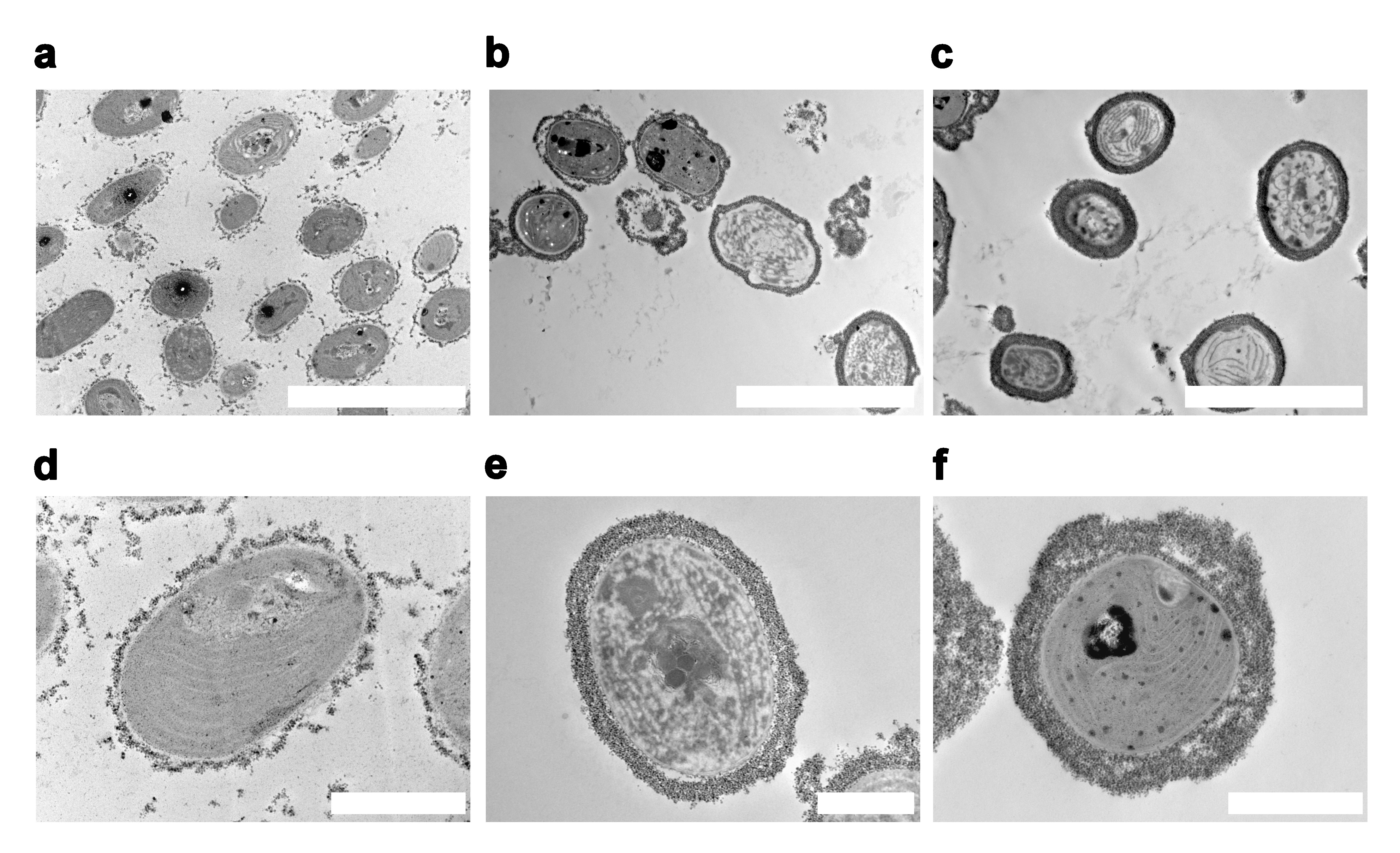


**Supplementary Figure 10. Structure of cyanobacteria encapsulated in cell-contacting shells with different thickness.** (**a-c**) TEM micrographs of cyanobacteria encapsulated in cell-contacting shells with different thickness of ~100 (**a**), ~200 (**b**) and ~300 nm (**c**). Scale bars, 3 μm. (**d-f**) TEM micrographs of cyanobacterium encapsulated in cell-contacting shell with different thickness of ~100 (**d**), ~200 (**e**) and ~300 nm (**f**). Scale bars, 1 μm.


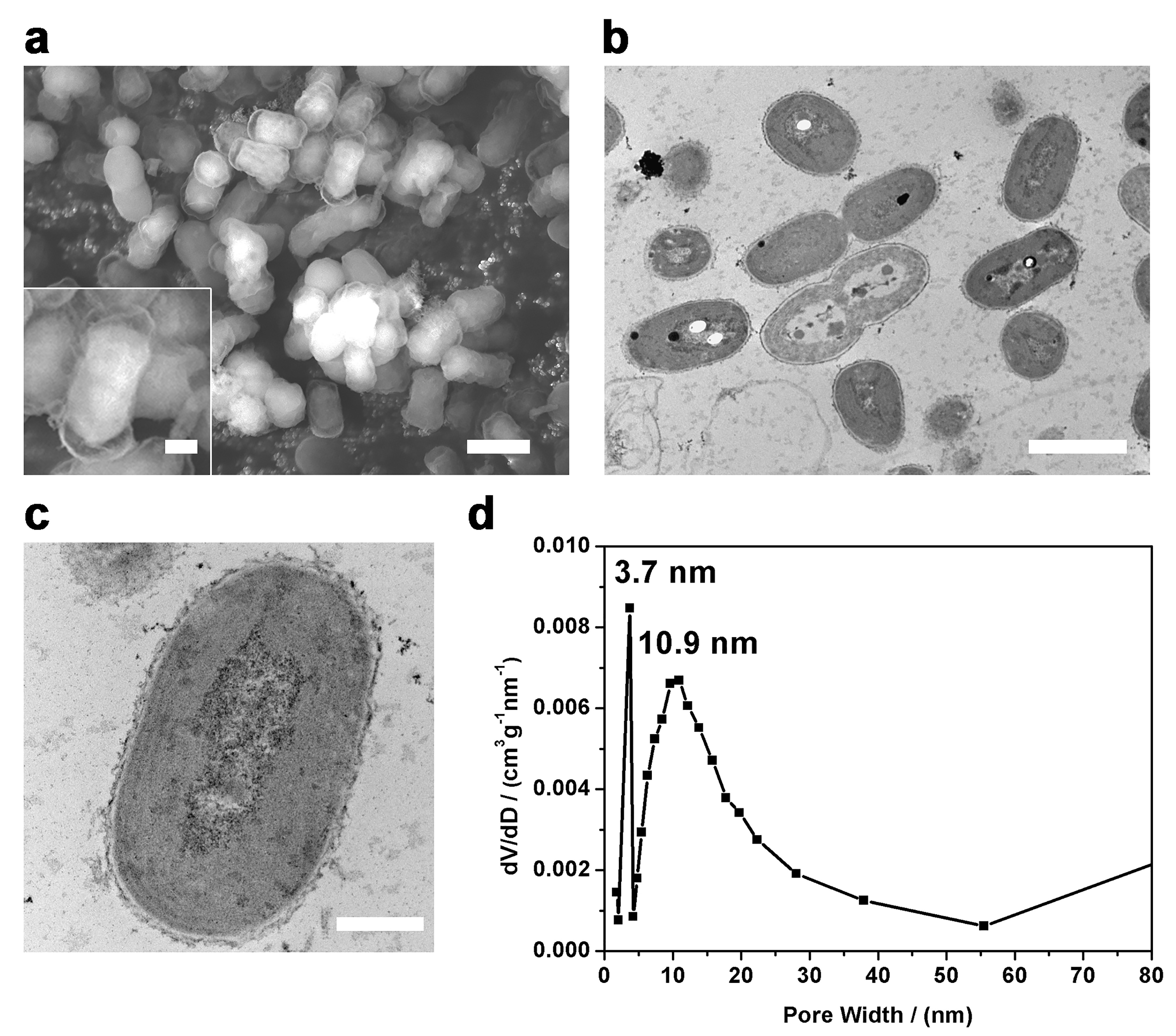


**Supplementary Figure 11. Characteristics of cyanobacteria encapsulated in disordered shells.** (**a**) SEM micrograph of cyanobacteria encapsulated in disordered shells. Scale bar, 2 μm; scale bar in the inset image, 500 nm. (**b**) TEM micrograph of cyanobacteria encapsulated in disordered shells. Scale bar, 2 μm. (**c**) TEM micrograph of a cyanobacterium encapsulated in a disordered shell. Scale bar, 500 nm. (**d**) Pore size distribution, peaking at 3.7 and 10.9 nm, of the disordered shells employed for comparison, determined using BJH analysis of nitrogen adsorption-desorption isotherms.


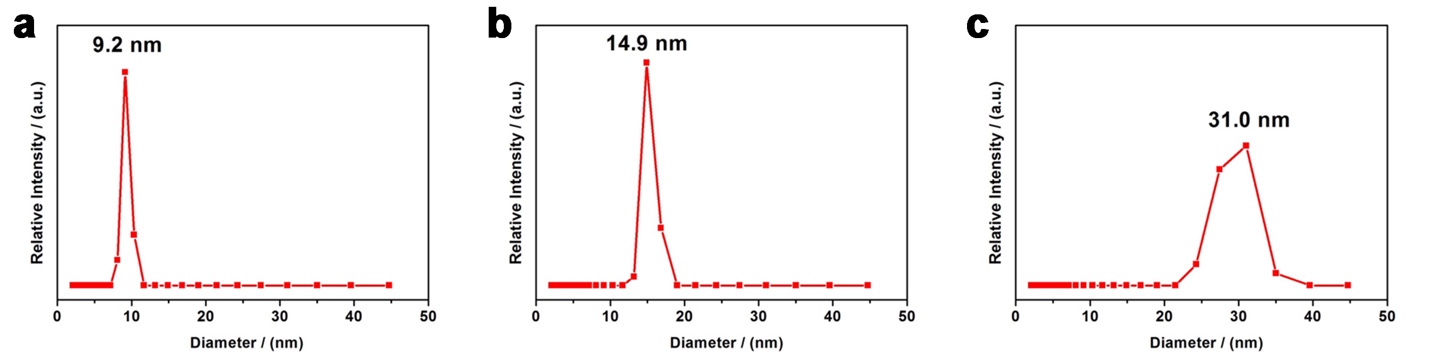


**Supplementary Figure 12. Different sized nanoparticles for cell encapsulation.** Diameter distributions of the silica nanoparticles used in this study for encapsulating cyanobacteria, obtained using dynamic light scattering. Particle diameters were taken as the diameter yielding the highest intensity as indicated in each graph.


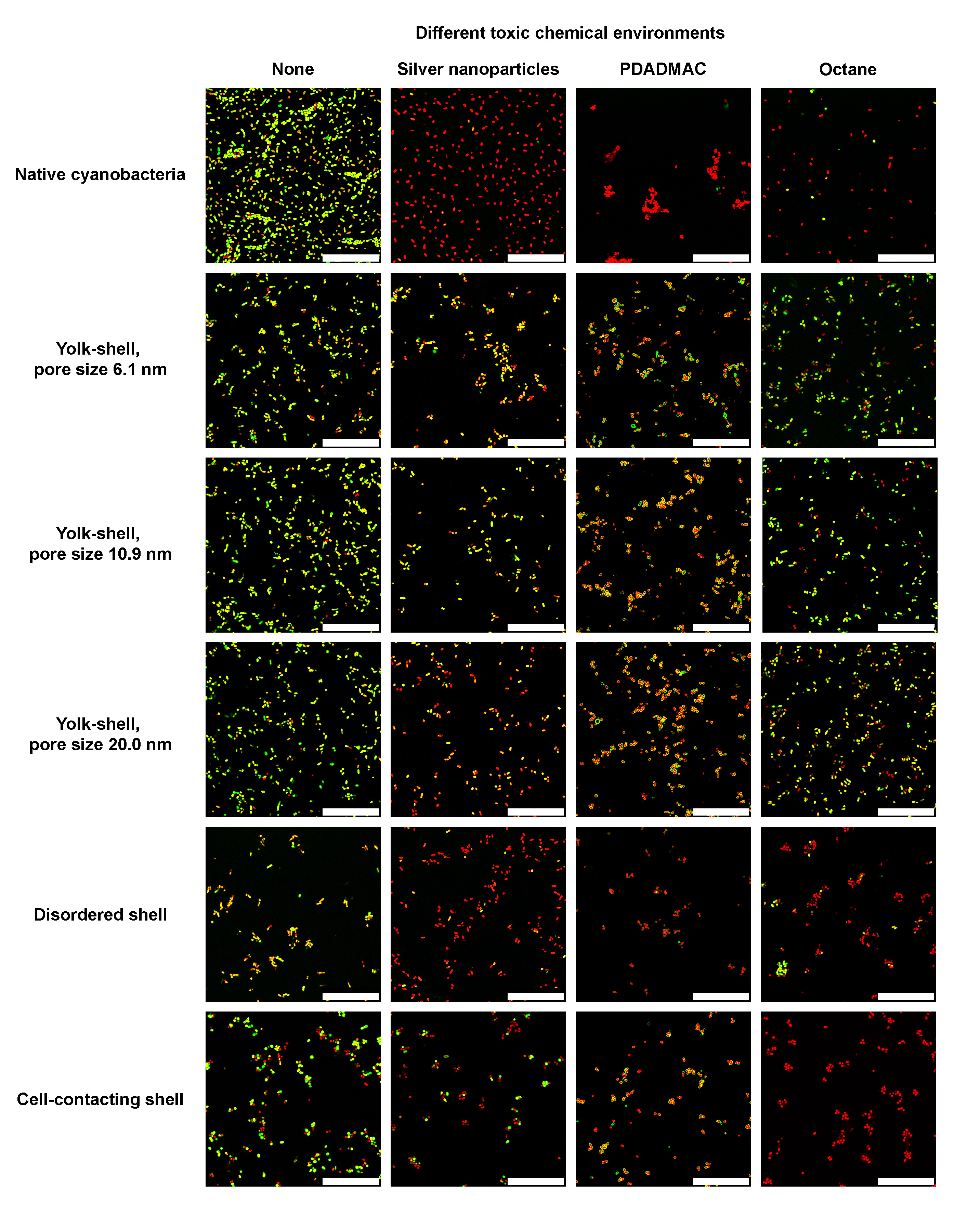


**Supplementary Figure 13. Survival ability of cyanobacteria in different toxic chemical environments.** Merged CLSM micrographs of FDA stained native cyanobacteria and cyanobacteria encapsulated in ordered yolk-shells with different pore size, in disordered shells and in cell-contacting shells after 1 day in the culture medium, with or without supplementation with silver nanoparticles, PDADMAC or octane. Greenish-yellowish fluorescent bacteria are alive, while the ones showing red-fluorescence are dead. Scale bars, 50 μm.


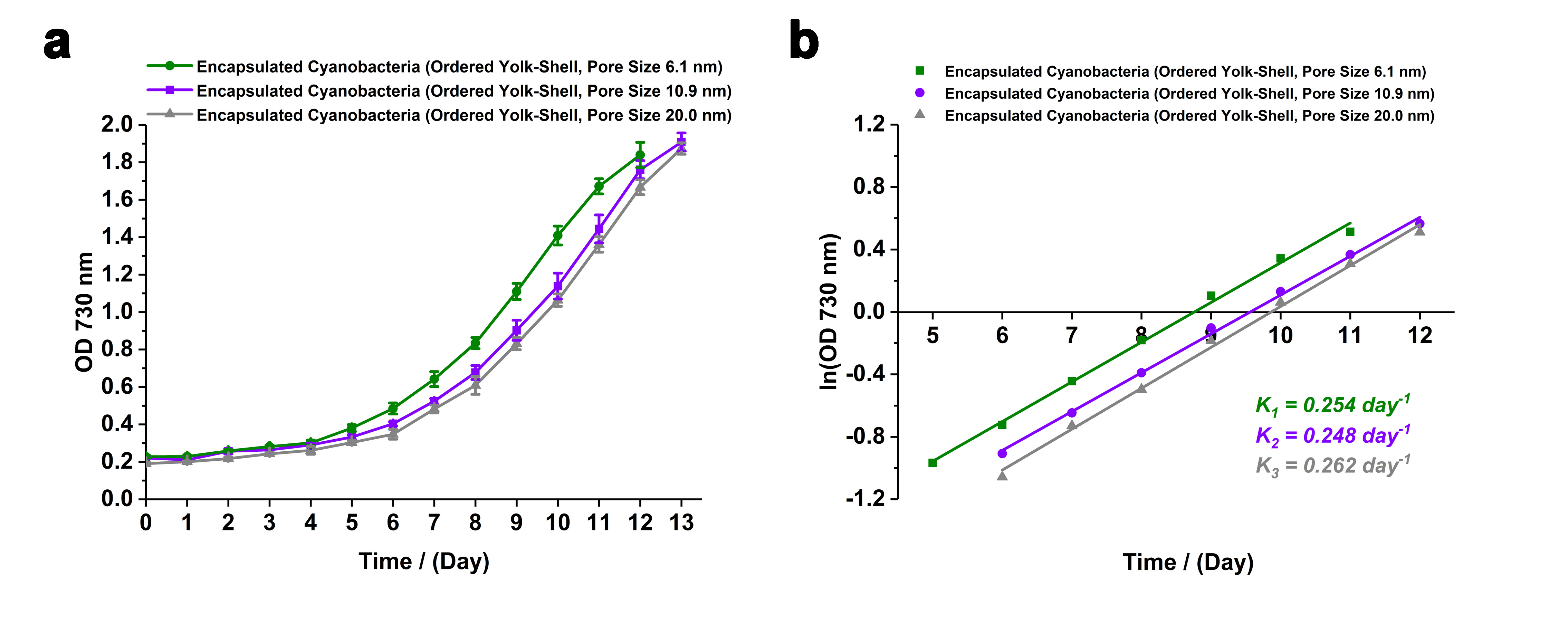


**Supplementary Figure 14. Yolk-shell influence on the cyanobacteria proliferation.** Optical densities (OD) at 730 nm of the cyanobacteria encapsulated in ordered yolk-shells with different pore size in growth medium as a function of time, plotted linearly (**a**) and logarithmically (**b**)*.* Error bars indicate standard deviations over three independent cultures.


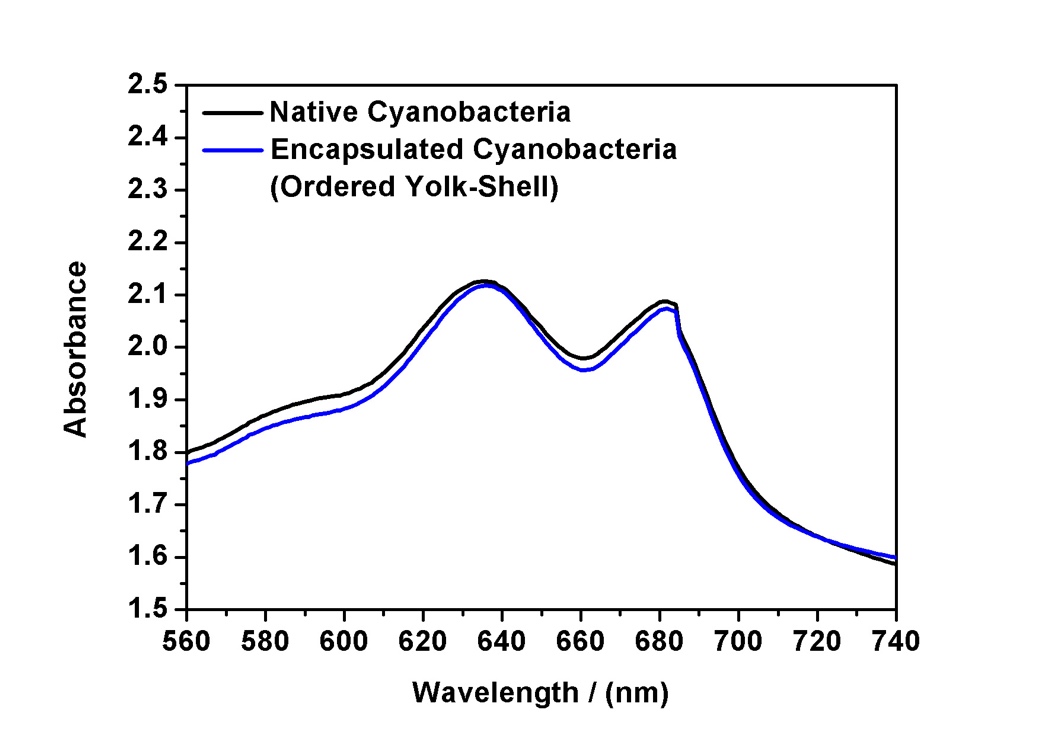


**Supplementary Figure 15. Visible spectrum of cyanobacteria.** Visible spectrum of native cyanobacteria and cyanobacteria encapsulated in a hierarchically-ordered ordered yolk-shell made with 14.9 nm colloidal nanoparticles.


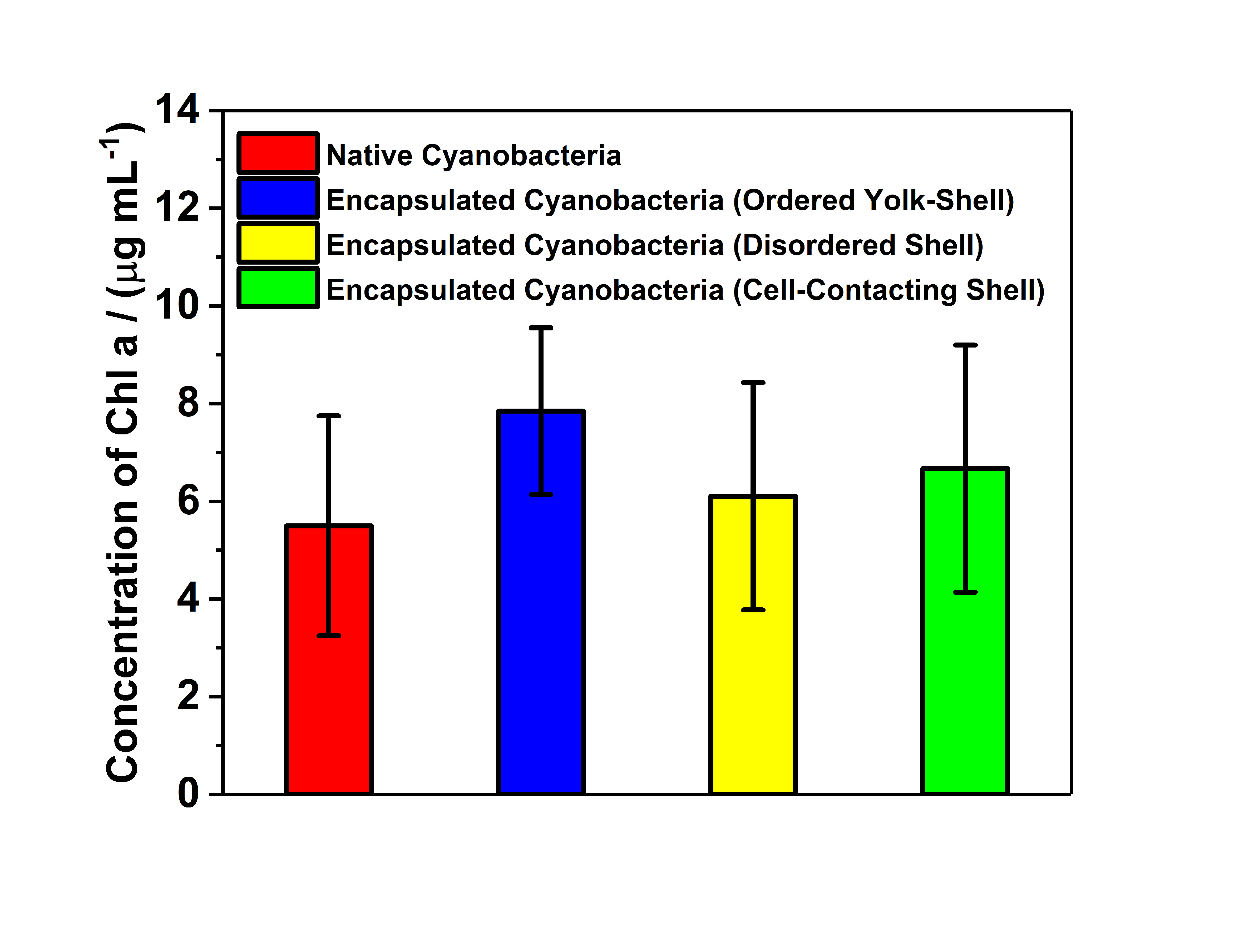


**Supplementary Figure 16. Concentration of *chlorophyll a*.** Concentration of *chlorophyll a* in native cyanobacteria, and cyanobacteria encapsulated in ordered yolk-shells (pore size of 10.9 nm), in disordered shells and in cell-contacting shells. Error bars indicate standard deviations over three independent measurements with separate cyanobacterial cultures. There is no significantly difference between native cyanobacteria and all kinds of encapsulated cyanobacteria at p < 0.05 (ANOVA).


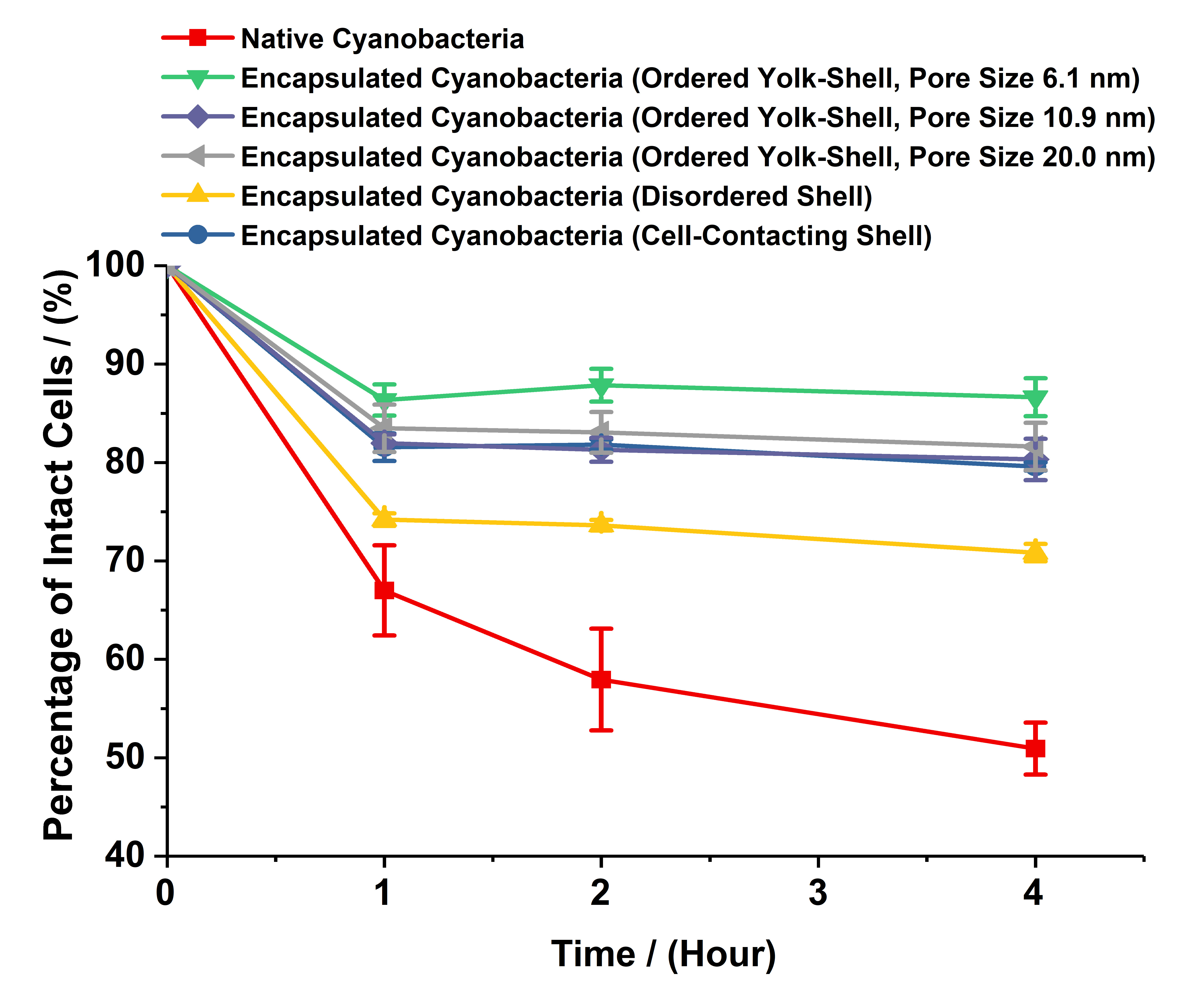


**Supplementary Figure 17. Effect of lysozyme on cyanobacteria.** Cell disintegration of native cyanobacteria and cyanobacteria encapsulated in ordered yolk-shells with different pore size, in disordered shells and in cell-contacting shells exposed to lysozyme-containing solution at 37 ^o^C as a function of time. The ratio of cell disintegration was followed over time by calculating the percentage of the real-time OD_730 nm_ to that prior to the lysozyme treatment. Error bars indicate standard deviations over three independent measurements with separate cyanobacterial cultures.


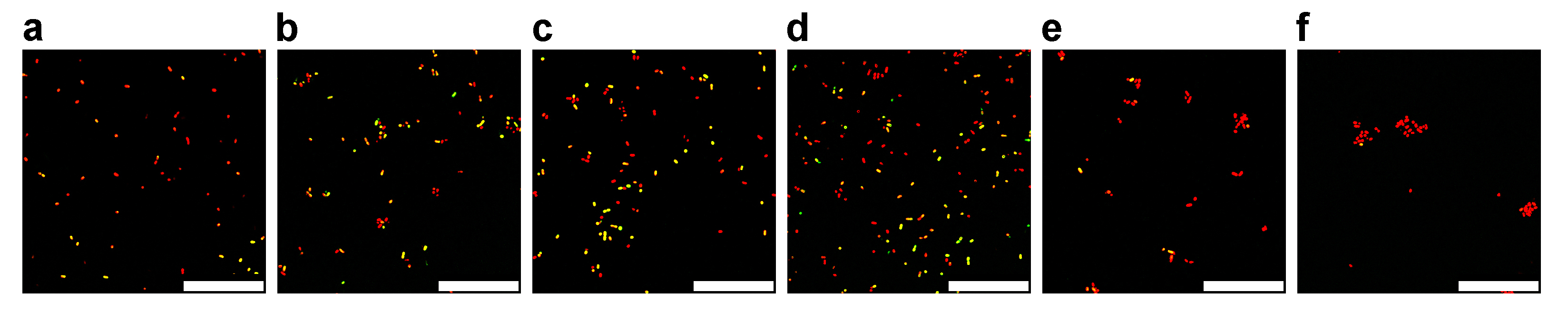

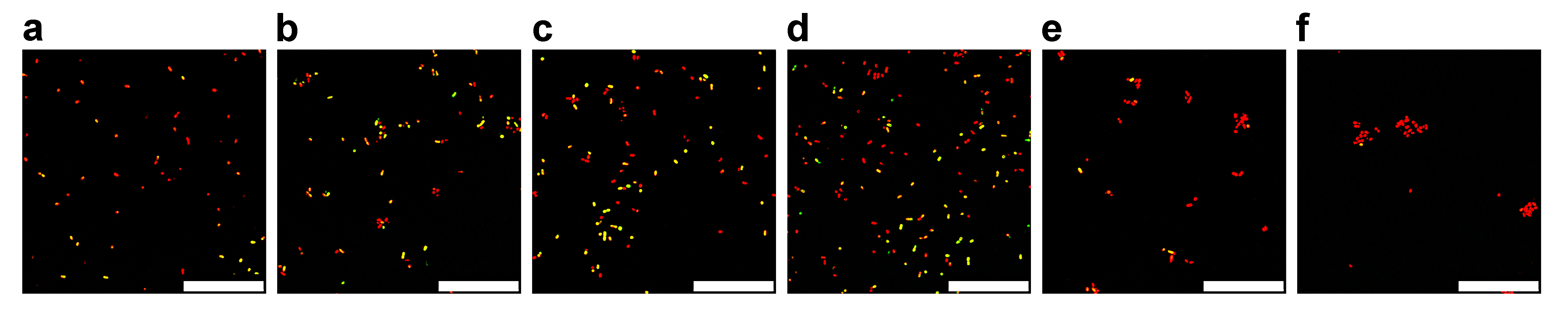


**Supplementary Figure 18. Thermal tolerance of cyanobacteria.** (**a-f**) Merged CLSM micrographs of FDA stained native cyanobacteria (**a**) and cyanobacteria encapsulated in ordered yolk-shells with a pore size of 6.1 (**b**), 10.9 (**c**) and 20.0 nm (**d**), in disordered shells (**e**) and in cell-contacting shells (**f**) after 1 h at 50 ^o^C. Greenish-yellowish fluorescent bacteria are alive, while the ones showing red-fluorescence are dead. Scale bars, 50 μm.


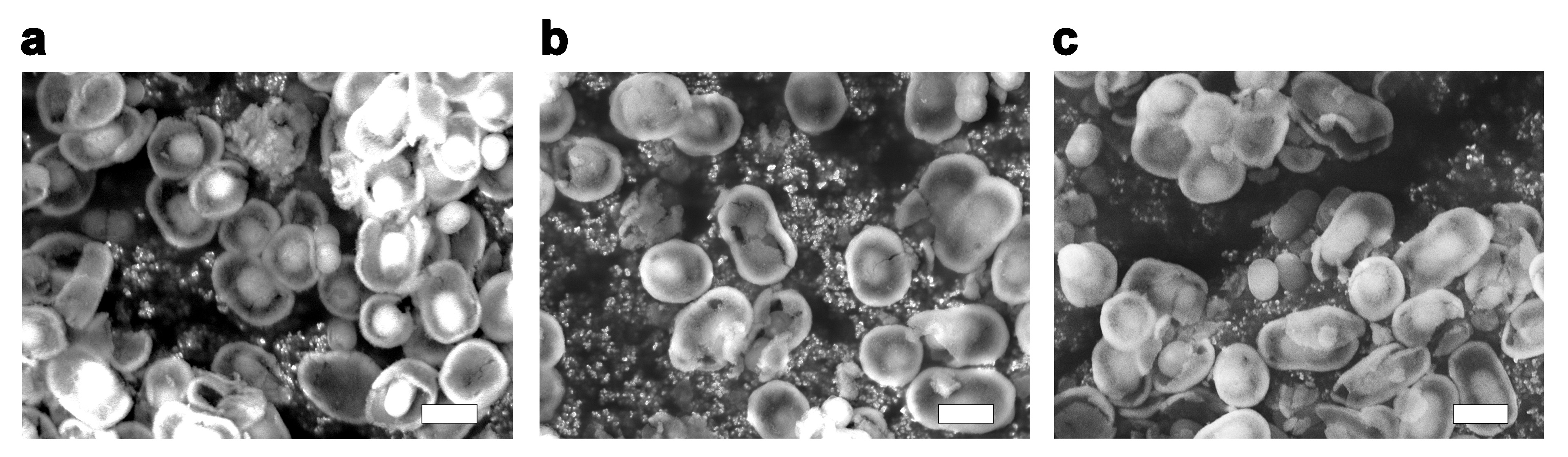


**Supplementary Figure 19. Stability of single cyanobacterium yolk-shell capsules.** SEM micrographs of single cyanobacterium capsules by protamine-assisted colloidal packing with nanoparticles of different diameters of 9.2 (**a**), 14.9 (**b**) and 31.0 nm (**c**) after 5-day culture. Scale bars, 2 μm.


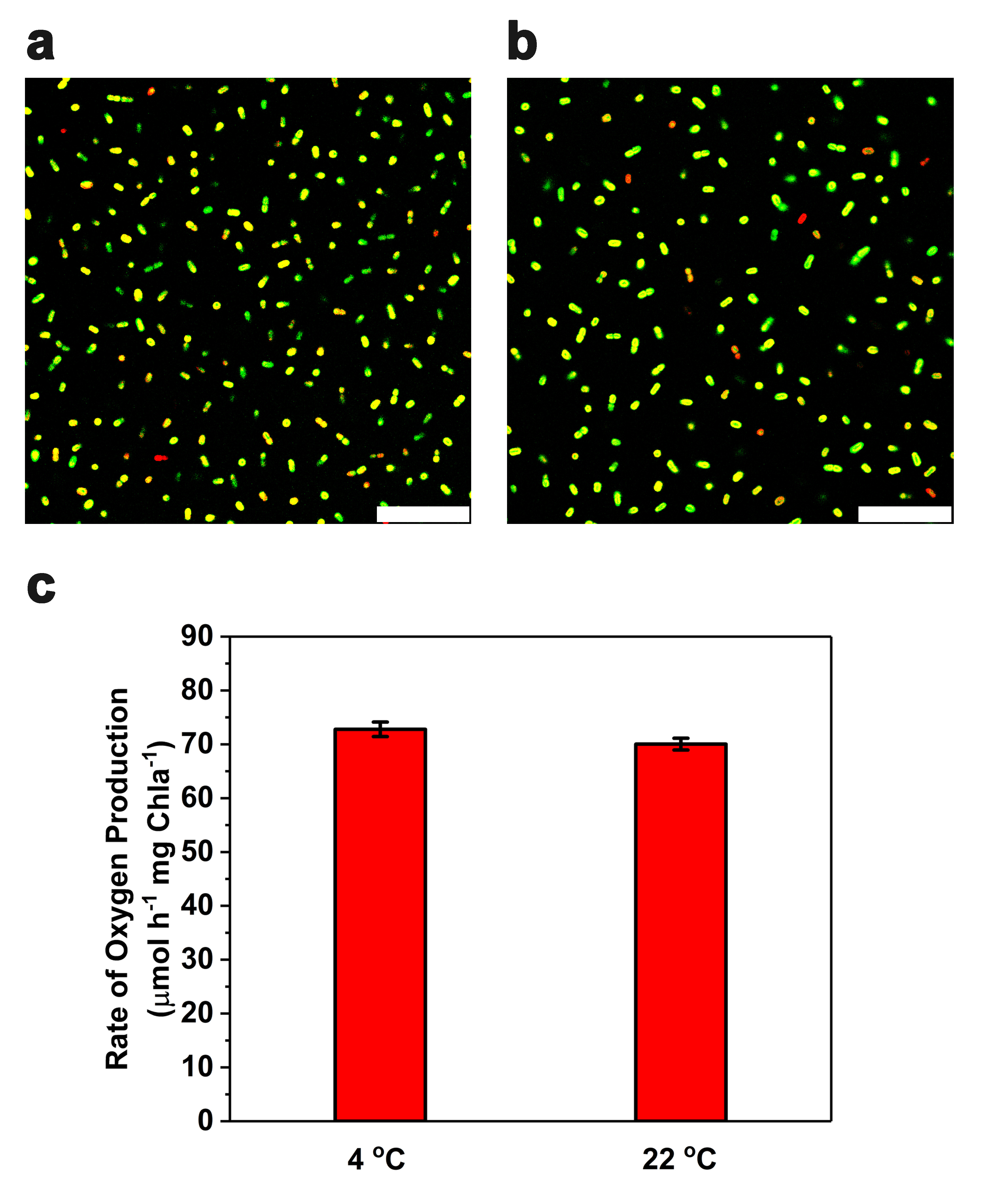


**Supplementary Figure 20. Influence of the centrifugation at different temperatures on the cyanobacterial viability.** (**a, b**) Merged CLSM micrographs of FDA-stained cyanobacteria after centrifugation (3620 *g* for 5 min) at 4 °C (**a**) or 22 °C (**b**). Greenish-yellowish fluorescent cyanobacteria are alive, while red fluorescent ones are dead. Scale bars, 25 μm. (**c**) Rate of oxygen production of native cyanobacteria at a photon flux density of 200 μE m^-2^ s^-1^ after centrifugation (3620 *g* for 5 min) at 4 or 22 °C. Error bars indicate standard deviations over three independent measurements with separate cyanobacterial cultures. There is no significant difference between two experimental groups at p < 0.05 (ANOVA).


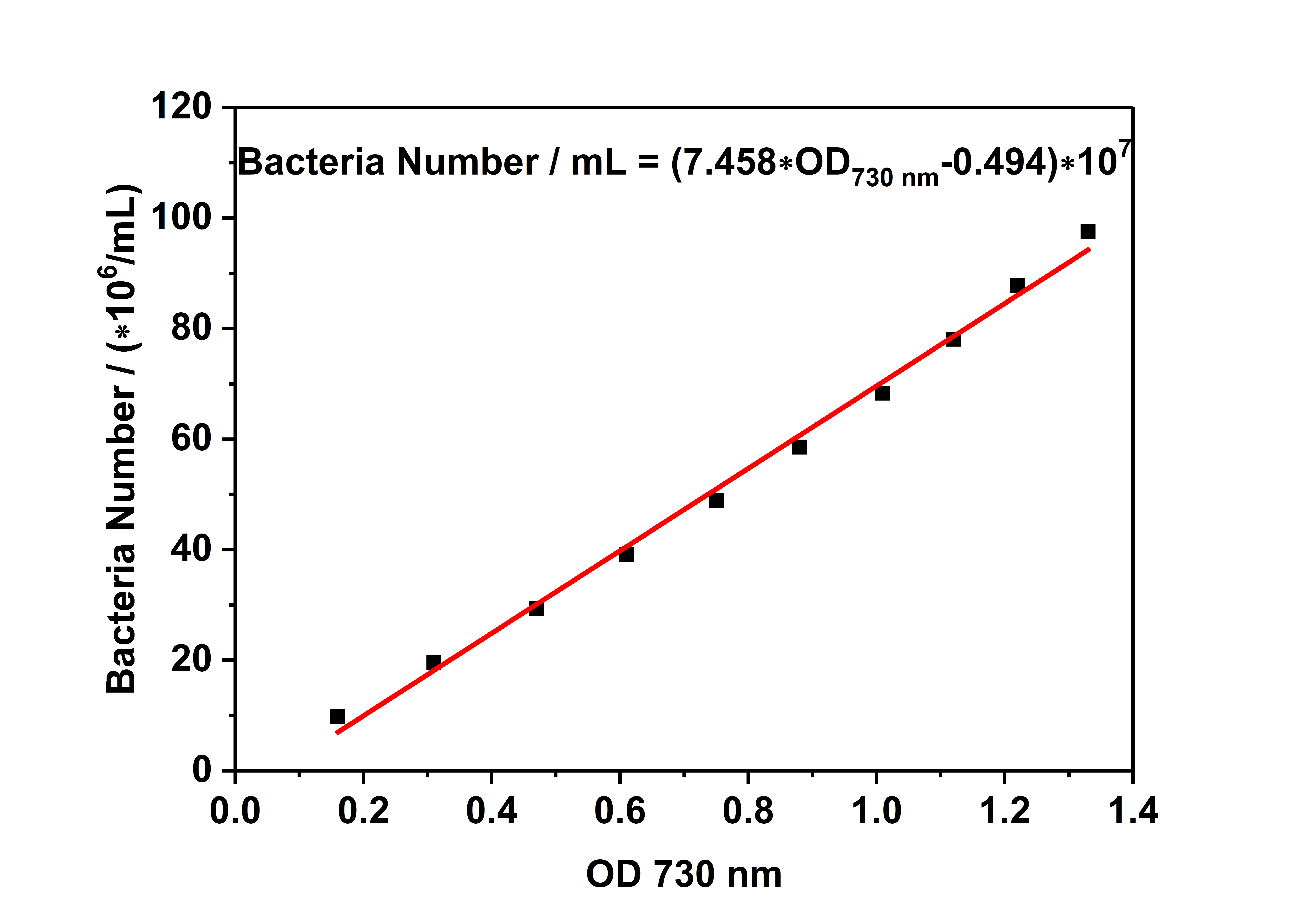


**Supplementary Figure 21. Bacterial numbers.** Calibration curve of optical density versus bacterial numbers. Calibration curve of OD_730 nm_ versus bacteria numbers.

**Supplementary Table 1. Parameters of cyanobacteria and encapsulated cyanobacteria.**

| **Encapsulation** | **Size of silica colloids**  **(nm)** | **Pore size**  **(nm)** | **Thickness of shells**  **(nm)** |
| --- | --- | --- | --- |
| Native cyanobacteria | n/a | n/a | n/a |
| Yolk-shell | 9.2 | 6.1 | ~120 |
|  | 14.9 | 10.9 | ~200 |
|  | 30.0 | 20.0 | ~250 |
| Disordered shell | n/a | 5 - 40 | ~30 |
| Cell-contacting shell | 14.9 | 7.3 | ~100 |

**Supplementary Table 2. Thermal tolerance of cyanobacteria.** Viability of native cyanobacteria and cyanobacteria encapsulated in ordered yolk-shells with different pore size, in disordered shells and in cell-contacting shells after 1 h at 50 ^o^C. Viability was expressed as the percentage of live cyanobacteria, visualized after FDA staining and observed using CLSM. All experiments were carried out with three separate samples with separately cultured cyanobacteria. The data represent percentage averages ± standard deviations obtained after counting a total of 200 cyanobacteria for each encapsulation and medium supplementation.

| **Encapsulation** | **Viability (%)** |
| --- | --- |
| Native cyanobacteria | 33 ± 9 |
| Yolk-shell, pore size 6.1 nm | 53* ± 8 |
| Yolk-shell, pore size 10.9 nm | 58* ± 10 |
| Yolk-shell, pore size 20.0 nm | 51* ± 7 |
| Disordered shell | 12*^,†^ ± 8 |
| Cell-contacting shell | 6*^,†^ ± 4 |

* Significantly different from native bacteria for each medium supplementation at p < 0.05 (ANOVA).

† Significantly different from cyanobacteria encapsulated in ordered yolk-shell for each medium supplementation at p < 0.05 (ANOVA).
